# Peptidome and Metabolome Profiling of Fermented Milk Products Reveal Accumulation of Bioactive Compounds During Cold Storage

**DOI:** 10.3390/cimb48090955

**Published:** 2026-09-19

**Authors:** Maxim S. Sheludchenko, Elena V. Kovalenko, Olesya O. Shoshina, Vera E. Odintsova, Stanislav I. Koshechkin, Olesya I. Volokh

**Affiliations:** 1 Health&Nutrition LLC, Krasnogorsk, Moscow Region 143421, Russia; 2 Nobias Technologies LLC, Sirius, Krasnodar Region 354340, Russia

**Keywords:** peptidome, fermented milk, kefir, metabolome, lipidome

## Abstract

This study assessed the metabolome and peptidome profiles of cow’s milk and four traditional fermented milk products produced using standard sets of starter cultures. Many of these microbe-generated molecules exhibit biological activities that can affect human health. We used non-targeted metabolomic methods to assess semi-quantified concentrations of four types of molecules in the final products: peptides; amino acids; long-, medium-, and short-chain fatty acids; and mono- and disaccharides and their derivatives. Ultra-performance liquid chromatography–mass spectrometry (UPLC-MS/MS) was performed on the peptidomes. For all other fractions, we used gas chromatography–mass spectrometry (GC-MS), with a method adapted to specific metabolite conditions. The metabolome and peptidome of 15 dairy milk products, including yogurt (Y), fermented milk (FM), kefir made with commercial cultures (K) and kefir made with grains (KG), were analyzed on days 7 and 14 of shelf life at +4 °C, and milk (M) samples with various fat content were used as a reference. A total of 348 peptides, 37 amino acids, 30 fatty acids, and 23 mono- and disaccharides were identified in the products. Among them, 41 putative bioactive peptides were annotated via in silico database mining, and together with branch-chained amino acids (BCAAs), orotic acid (vitamin B13), d-phenyllactic acid, 5-phenylvaleric acid and myo-inositol (vitamin B8), accumulated in fermented products during storage. Bioactivities were not experimentally validated in this study.

## 1. Introduction

Fermentation is an ancient way of preserving food and improving its shelf life [1,2]. In recent decades, fermented milk products have garnered considerable research attention owing to their numerous health benefits, such as the improved digestion and bioavailability of milk constituents, the inhibition of harmful gastrointestinal bacteria, the alleviation of lactose intolerance, and the effects of fermented milk products and the gut microbiome on brain activity; therefore, they are considered functional foods [3,4,5,6]. Metabolites reflect the quality of milk and dairy products in terms of their nutritional value and safety [7]. Notably, the identified differentially abundant metabolites are believed to play a crucial role in shaping the distinctive gustatory and olfactory characteristics of fermented milk products [8]. The metabolism of macronutrients is essential for the production of fermented milk products, such as yogurt, cheese, and kefir. The specific types and quantities of metabolized compounds can be affected by the microorganisms present in the starter culture. Factors such as raw milk, fat percentage, season, temperature, and geographical origin as well as contamination or adulteration during processing and transport may also alter the metabolite profiles produced by microorganisms [9,10,11,12].

In the case of proteins, fermentation involves microbial hydrolysis of the peptide bonds, which results in the release of oligopeptides and free amino acids. The initial amount of free amino acids and peptides in milk is limited and only sustains the starter culture for up to five generations [13]. Therefore, lactic acid bacteria (LAB) and yeasts employ a complex proteolytic system to degrade milk proteins. It has been observed that the proteolytic activity in milk is associated with the formation of polypeptides and oligopeptides through the action of bacterial peptidases that break down larger molecules, resulting in the production of smaller peptides and free amino acids [8]. Studies have also shown that bacterial proteases and peptidases remain active during the fermentation and storage of fermented milk, and these enzymes exhibit specificity for specific cleavage sites or sequences during proteolysis [14]. Amino acids may serve as building blocks for most volatile fatty acids, since they can help to make acids [15].

Fats in fermented food are broken down by lipolysis. The medium-length fraction of fatty acids appears to be utilized by microorganisms during their metabolic processes, thereby preventing their excessive accumulation [16,17]. The fraction of short-chain fatty acids is the most prominent component of fermented products [18]. This could be a consequence of lipolysis and glycolysis, and it is generally accepted that C2–C4 acids are produced by LAB and that C4–C20 acids are mostly produced by fat [15]. Through these mechanisms, the fat fraction undergoes transformations, leading to varied compositions of different fermented products. LAB are thought to have weak lipolytic activity compared to yeasts or other bacterial taxa such as *Pseudomonas*, *Flavobacterium,* and *Staphylococcus* [19,20]. However, in products such as cheese, with extended ripening times, the lipolytic activity of LAB contributes to flavor development and serves as a substrate for further reactions, leading to the formation of end-products [21]. *Lactococcus lactis,* as single started culture in sour cream, was shown to decrease the fat content due to lipase activity causing hydrolysis or possibly by milk oxidation associated with the autolysis of LAB [22]. Conjugated linoleic acid, as a bioactive fatty acid, is one of the desirable products of lipid metabolism in milk, but knowledge about its conversion remains limited [23]. Therefore, evaluation of the degree of lipolysis might help to determine the best strains to use as starter cultures.

Carbohydrates are other potential substrates in milk that can be readily utilized by microorganisms. LAB secrete various extracellular and capsular polysaccharides. Complex carbohydrates play a crucial role in determining the unique textural properties of various fermented milk products, influencing their viscosity, creaminess, and mouthfeel. Dairy oligosaccharides are composed of 3–20 monosaccharides [24]. Typically, only 30% of lactose is converted into glucose and galactose by β-galactosidase during milk fermentation [25]. Glucose is metabolized by bacteria into lactic acid through glycolysis [26].

Metabolomics involves a comprehensive analysis of both the quantity and characteristics of microbial metabolites produced during fermentation. Therefore, it has revolutionized our understanding of microbial metabolism, genetic characteristics, and protein synthesis. By examining these metabolites, researchers have gained insight into the intricate biochemical pathways and chemical transformations that occur during this process. Thus, metabolomics serves as an invaluable tool for tailoring fermented food products, producing improved functionality and health benefits [27]. Some peptides possess specific physiological functions that are beneficial to human health. Depending on the peptide composition, they exhibit antihypertensive, antioxidant, bacteriostatic, opioid-like, anti-inflammatory, antiproliferative, antithrombotic, hypolipidemic, hypocholesterolemic, and metal-scavenging properties [28]. These peptides are thermostable, and their resistance to gut proteases helps ameliorate hypertension by inhibiting angiotensin enzymes in the aorta. This effect has been demonstrated in rats [29], and later in humans [28]. Peptides and free amino acids are taken up by enterocytes of the small intestine, and short peptides consisting of two to three amino acids are absorbed [30,31].

However, the existing literature primarily focuses on changes during the fermentation process itself, while insufficient attention has been given to “post-fermentation” metabolic remodeling of industrial products during cold-chain distribution and shelf life. In the current study, we focus on two aims: evaluation of the potentially beneficial major organic compounds of fermented dairy products in contrast to raw milk and assessment of the decomposition dynamics of compounds with high-molecular weight during 7 and 14 days of cold storage.

## 2. Materials and Methods

### 2.1. Sample Description, Storage Characteristics and Aliquots Taken for the Analysis

The current study was carried out on a range of commercial fermented milk drinks (*n* = 6) vs. normalized milk (*n* = 3): kefir made with wild kefir grains (KG), with 1.2% and 2.5% fat (*n* = 2); kefir drink (K) made with commercial cultures of *Lactobacillus acidophilus*, *Lactococcus lactis*, *Lactococcus mesenteroides*, *Bifidobacterium animalis* and *Debaryomyces hansenii*, with 0.1% and 1% fat (*n* = 2); fermented milk (FM) with 2.5% fat (*n* = 1); probiotic yogurt (Y) with 1.7% fat (*n* = 1); and normalized milk (M) with 1.2%, 2.5% and 3.2% (*n* = 3) fat, used as a reference without fermentation. All products were supplied by the dairy company Health&Nutrition LLC (Krasnogorsk, Russia). The concentrations of LAB in the starter cultures were at least 1 × 10^10^ CFU/g, *B. animalis* at least 1 × 10^8^ CFU/g, and yeast at least 1 × 10^4^ CFU/g. K contained a single yeast strain, KG contained 10 yeast strains in the starter cultures, and FM and Y did not contain any yeast in the starter cultures. Each product with a range of fat content (except milk) was sampled at 7 and 14 days of storage at +4 °C. To study the decomposition dynamics of the substances, we used these two time points for the shelf life (D + 7 vs. D + 14). We selected days D + 7 and D + 14 as sampling points due to KG having only 16 days of shelf life. Most dairy products are delivered to supermarkets around D7 owing to logistics, since products must be passed by QC after a few days of storage and transported by cold chain to regional shops. The total number of dairy products analyzed was *n* = 15 (Table 1).

For each dairy product, 250 mL of sample in five aliquots of 50 mL was used for further analysis. The obtained aliquots were stored at −35 °C to avoid any freeze–thaw cycles until brought into the laboratory.

**Figure 1:**
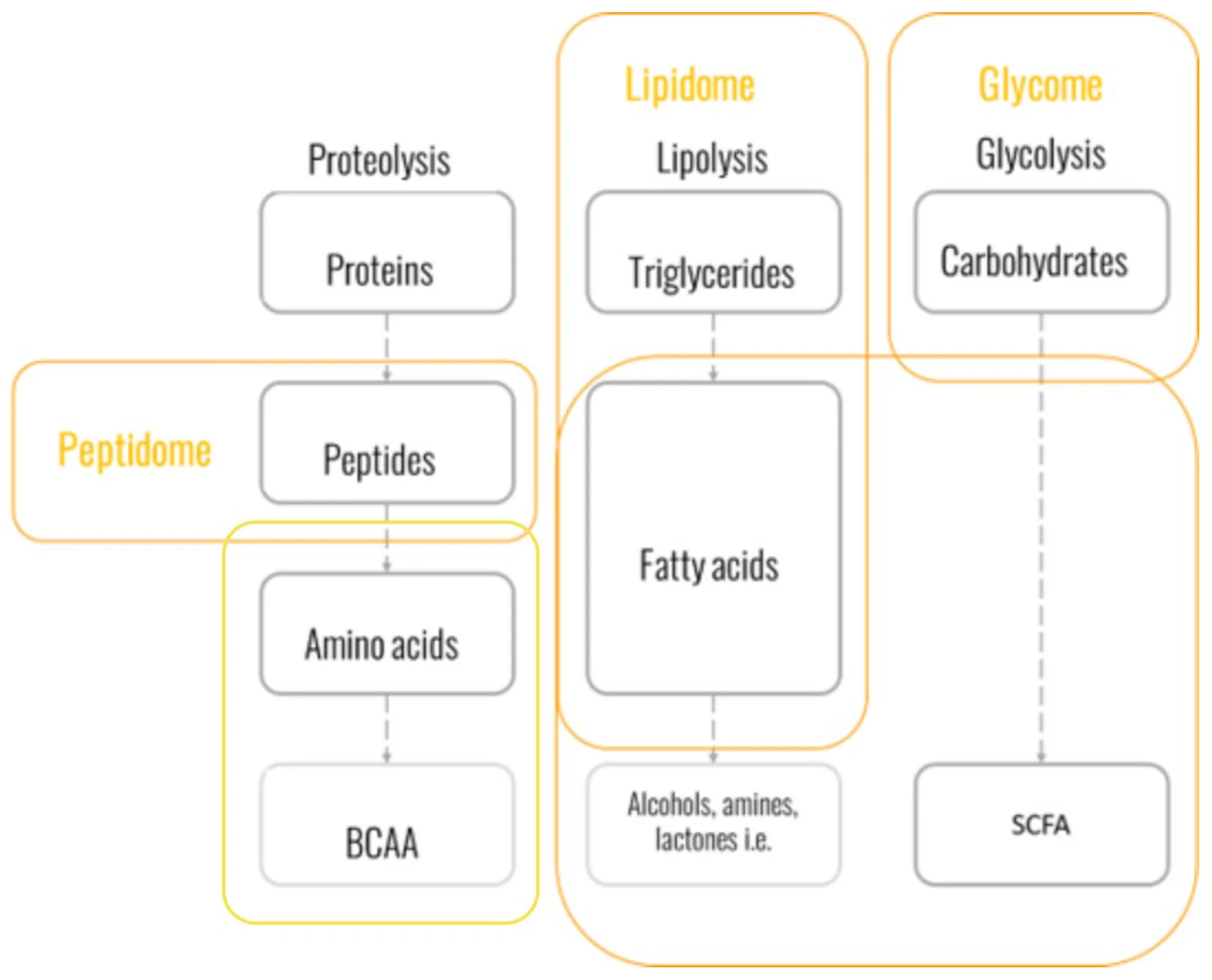
Schematic representation of methods (peptidome, free amino acid analysis, lipidome, and glycome) applied in this study for peptidome and metabolome profiling; orange color indicates “omics” panels combining few analytical methods.

Metabolite analyses were used to evaluate the peptidome and four varieties of untargeted metabolomes, in order to evaluate different fractions of organic compounds (fatty acids, short fatty acids, amino acids, and sugars) (Figure 1). In addition to metabolite analysis, biochemical methods were used to determine total protein in the sample and total free peptides as part of the sample preparation, as well as total fat (triglycerides). The description of the methods used in this study is presented below.

### 2.2. 16s rRNA and ITS Amplicon Sequencing Analysis of KG Samples

Samples of kefir grains and the white mass of KG were received in triplicate (20 mL each) in a frozen format and preserved at −20 °C until processed. DNA was extracted using an internal protocol with the aid of a MagnaPure automatic extractor (Roche Life Sciences, Basel, Switzerland). Two sets of primers were used: the first pair, S-D-Bact-0341-b-S-17, 5′-CCTACGGGNGGCWGCAG-3′ and S-D-Bact-0785-a-A-21, 5′-GACTACHVGGGTATCTAATCC-3′, capturing the region for 16s rRNA with an amplicon size of 464 bp [32] of the bacterial taxa; and the second pair, ITS3_KYO2, 5′-GATGAAGAACGYAGYRAA-3′ and ITS4, 5′-TCCTCCGCTTATTGATATGC-3′, capturing the region for ITS with an amplicon size of approximately 500 bp [33] of the fungi. An artificial mock community, composed of 25 *Lactobacillus* and 75 *Bifidobacterium* strains, was included as a positive control. Samples were sequenced at the Biopolis S.L. CIFB (Valencia, Spain) facility using the MiSeq Platform (Illumina, San Diego, CA, USA) with a 300PE combination.

For bioinformatic analysis, pair-end raw sequences were merged in order to obtain the complete sequence with ‘pear v0.9.6’. The amplification primers were trimmed by ‘cutadapt v 1.8.1’ with default parameters. Sequences less than 200 nt were not further considered for the analysis, and a quality filter was applied to delete sequences of poor quality. Bases in extreme positions that did not have Q20 (99%) of well-incorporated bases in the sequencing step or a more pared score were removed; sequences whose quality mean did not surpass the Q20 threshold, as a mean quality of the whole sequence, were also deleted. The resulting sequences were inspected for PCR chimera constructs that may have occurred during different experimental processes, and chimeras were removed. In order to reduce the complexity of the annotation, sequences with 97% similarity were clustered into a single cluster using the program ‘cd-hit’, so only the representative sequences needed to be annotated, and the results were applied to the cluster of sequences represented by the analysis. Each of the clean clustered sequences was compared against the built rRNA database using a BLAST +2.6.0 local alignment approach based on the publicly available source https://ftp.ncbi.nlm.nih.gov/blast/db/ (accessed on 20 February 2017), in order to associate each of the clusters with one taxonomical group from the database.

### 2.3. Peptidome Analysis

#### 2.3.1. Sample Preparation for Peptidome by Free Peptide Extraction

Three biological replicates of 10 mL from 50 mL thawed aliquot were lyophilized in 50 mL tubes. Thereafter, 20 µg of lyophilized dried mass from each sample was resuspended into 1 mL of 3% acetonitrile (ACN) and 0.1% of trifluoroacetic acid (TFA) (Sigma-Aldrich, Darmstadt, Germany, cat#1.08178) in milli-Q water (Millipore ZLXE0030RU, Molsheim, France) and shaken at 2000 rpm (ELMI S-3M. A10, Riga, Latvia) for 30 min. After centrifugation at 1100 rpm for 30 min, the supernatant was collected and filtered through a 0.22 µm 25 mm FLL/mIS CA ST syringe filter (GVS, Bologna, Italy). The obtained extracts were further purified using an Oasis C18 column (Waters, Framingham, MA, USA). The eluates were dried under vacuum and diluted in 30 µL milli-Q water containing 3% ACN and 0.1% TFA. The peptide concentration was determined using the BCA Assay Kit (Sigma-Aldrich, cat #71285-M) for all techniques.

#### 2.3.2. Ultra-Performance Liquid Chromatography–Mass Spectrometry Analysis (LC/MS)

Free peptide extracts were analyzed separately using a nano-ESI Orbitrap Q Exactive HF-X mass spectrometer (Thermo Fisher Scientific, Waltham, MA, USA) coupled to a nano-flow high-pressure chromatograph (UPLC Ultimate 3000, Germering, Germany) with a reverse-phase C18 column 100 µm × 300 mm (Thermo Fisher Scientific, USA). For sample analysis, a gradient of 0–40% buffer B (buffer A: 0.1% formic acid in deionized water (18Mohm) and buffer B: 80% acetonitrile and 0.1% formic acid in deionized water) was used with a flow rate of 0.25 µL/min. Emitter needle voltage was set to +2.2 kV, RF voltage at S-lens was 65, and capillary temperature was 250 °C. MS and MS/MS spectra were acquired at resolutions of 60,000 and 30,000, respectively. Charge accumulation cut-off levels were 2e6 for MS and 2e5 for MS/MS mode, with maximum ion accumulation times of 45 and 50 ms, respectively. The protocol for the assessment of relative peptide concentrations is described in Supplementary Material File S1 with calibration curves presented in Figure S1. Samples KG2.5_D7 and M2.5 were analyzed in triplicate, and peptide identities based on peak intensity variations were 1.25% and 15.83%, respectively (see Table S1). The other fermented products were measured once based on very low variation of the KG2.5_D7 sample, to reduce the cost of the total study.

### 2.4. Lipidome, Glycome and Free Amino Acid Analysis

#### 2.4.1. Total Triglyceride Analysis

Sample preparation was performed using two biological replicates. Seven grams of each sample was thawed with 7 mL of concentrated HCl, mixed, and kept in an oven at 90 °C for 1 h. After cooling to room temperature, samples were mixed with 2 mL methanol and 20 mL hexane and shaken vigorously using a vortex, and the hexane layer was collected in a new tube. The remaining water fraction of the same sample was mixed with 2 mL methanol and 20 mL diethyl ether was added; the sample was shaken vigorously using a vortex, and the obtained extract was combined with hexane extract. The remaining watery fraction was mixed with 500 mg NaCl, extracted with 20 mL hexane and combined with other extracts. The combined final extracts were dried in an oven at 90 °C to obtain a constant weight. The obtained fat fraction was weighted on analytical balances. The percentage of fat in the original sample was calculated based on the difference between the initial weight and the weight of the dried sample.

#### 2.4.2. Free Fatty Acid Analysis

Thawed samples (20 g) from two biological replicates were mixed with 10 g of NaCl on an orbital shaker. Once the salt was completely dissolved, 25 mL of ACN and 1 mL of 10% HCl were added to obtain pH 1–2. Samples were incubated at room temperature with 450 rpm for 10 min, followed by centrifugation at 3500 rpm for 10 min. The supernatant was collected, mixed with 20 mL of water, and neutralized with 5M KOH to obtain a pH of 5–6, and the obtained mixture was evaporated to 20 mL. Next, 5M KOH was added to obtain a pH of 10, and 25 mL of hexane was added. The mixture was incubated at room temperature on a shaker at 450 rpm for 10 min and then centrifuged at 3500 rpm for 10 min. The bottom aqueous layer was collected, acidified with 10% HCl to obtain a pH of 2–2.5, mixed with 25 mL dichloromethane, and incubated at room temperature with 450 rpm for 10 min, followed by centrifugation at 3500 rpm for 10 min. The bottom layer was collected, and the ACN layer was collected and evaporated to a minimal volume, transferred into a glass vial, and dried under nitrogen gas (Microvap, Oranomation, Berlin, MA, USA) at 60 °C. The residue was derivatized with 70 µL of N-Methyl-N-(trimethylsilyl)-trifluoroacetamide (MSTFA) (Sigma-Aldrich, cat # 69479), incubated at 70 °C for 30 min, mixed with 400 µL of ethyl acetate and analyzed by GC-MS.

#### 2.4.3. Free Carbohydrate (Mono- and Disaccharide) Analysis

Thawed samples in two biological replicates (5 g fermented milk or 1 g milk) were extracted with ACN and acidified with 10% HCl to obtain a pH of 1–2 by mixing on an orbital shaker at room temperature for 10 min. Protein fractions were separated by centrifugation at 3500 rpm for 10 min. The bottom aqueous layer (1 mL) was transferred to a glass vial and neutralized with 5M KOH to obtain a pH of 5–6, evaporated to a minimal volume with a heater block, and completely dried by adding isopropanol under a nitrogen flow at 60 °C. After, the pellet was derivatized with 200 µL MSTFA and incubated under a closed cap at 70 °C for 20 h. The derivatized carbohydrates were mixed with 400 µL ethyl acetate, vortexed, transferred into a 1.5 mL tube and centrifuged at 13,500 rpm for 10 min at room temperature. The supernatant was then placed into a glass vial for GC-MS analysis.

#### 2.4.4. Free Amino Acid Analysis

Sample preparation was performed independently in two biological replicates. Ten grams of NaCl were added to 20 g of the thawed sample, and the mixture was stirred on a shaker until the salt was completely dissolved. Next, 25 mL of ACN was added and acidified with 1.0 mL of 10% HCl to obtain a pH of 1–2. The mixture was incubated at room temperature with stirring at 450 rpm for 10 min and centrifuged at 3500 rpm for 10 min. The upper layer with ACN was mixed with 15 mL of water and 5M KOH solution to obtain a pH of 5–6 and then evaporated to 20 mL. A solution of 5M KOH was added to obtain a pH of 10, 20 mL of hexane was added, and the mixture was incubated at room temperature with stirring at 450 rpm for 10 min, followed by centrifugation at 3500 rpm for 10 min.

The bottom aqueous layer was collected and acidified with 10% HCl to pH 2–2.5. Next, 25 mL dichloromethane was added and incubated at room temperature at 450 rpm for 10 min, followed by centrifugation at 3500 rpm for 10 min. The collected upper aqueous layer was evaporated to a minimum volume in a glass vial, five times the volume of isopropanol was added, and the mixture was dried under a nitrogen flow at 60 °C. The dried sample was mixed with 100 μL of MSTFA, and the reaction was carried out under a closed cap at 70 °C for 4 h and then mixed with 400 µL of ethyl acetate. Finally, the mixture was centrifuged at 13,500 rpm for 10 min at room temperature, and the supernatant was transferred to a 1.5 mL vial for GC/MS analysis.

#### 2.4.5. Gas Chromatography with Mass Spectrometry (GC-MS)

Compound profiles for the samples were acquired with use of an Agilent 8890 GC System (Agilent, Singapore) with the mass-spectrometry detector Agilent 5977B GC/MSD, equipped with an Agilent 7693 autosampler. The analytes were separated using an HP-5MS column (30 m × 0.25 mm × 0.25 µm) (19091S-433UI-KEY, Agilent, Folsom, CA, USA) under helium at a pressure of 95 kPa and a gas flow of 1.3 mL/min. The injection volume was 1 µL, with conditions as specified for the class of compounds.

For free fatty acids, the following conditions were used: injection at 250 °C without flow division. Initial oven temperature was 100 °C for 5 min, with a final temperature of 280 °C, a ramping rate of 4 °C/min, a dwell time of 12 min, and overall analysis of 62.0 min. The MS detector temperature was 280 °C, with MS in full scanning mode in the range of 40–700 m/z with a frequency of 12.8 scans/s.

For free carbohydrates, the following conditions were used: injection at 270 °C, with a flow separation of 5:1. The initial oven temperature was 100 °C for 1 min, with a final temperature 300 °C and a ramping rate of 25 °C/min; dwell time was 5 min at 250 °C with an overall time of analysis of 14.0 min. The MS detector temperature was 280 °C, with MS in full scanning mode in the range of 40–500 m/z with a frequency of 12.8 scans/s.

For free amino acids, the following conditions were used: injection at 270 °C, without gas flow separation. The initial oven temperature was 80 °C for 7 min, with a final temperature of 100 °C; 10 min dwell time at 100 °C with a ramping rate of 3 °C/min up to 120 °C; 10 min dwell time at 120 °C with a ramping rate of 3 °C/min up to 140 °C; 10 min dwell time at 140 °C, with a ramping rate of 3 °C/min up to 160 °C, 10 min stop-over at 160 °C, and a ramping rate of 3 °C/min up to 180 °C; 1 min dwell time at 180 °C, with a ramping rate of 4 °C/min up to 280 °C; and dwell time 4 min at 280 °C, with an overall analysis time of 110.3 min. The MS detector temperature was 280 °C, with MS in full scanning mode in the range of 40–700 m/z with a frequency of 12.8 scans/s.

Peaks were analyzed using MassHunter GC/MSD ChemStation version 11.0 software. Untargeted metabolites were identified by comparing the spectra of each peak using the NIST library collection (NIST, Gaithersburg, MD, USA). The linear index difference maximum tolerance was set to 10, and the minimum matching spectra library search was set to 85% (level 2 identification, as described by the Metabolomics Standards Initiative [MSI]) [34].

Each metabolite peak area was normalized to the internal standard of 2-iso-propylmalic acid (Sigma-Aldrich, cat #333115) for carbohydrates and amino acids and Supelco37 standard (Sigma-Aldrich, cat #CRM47885) for free fatty acids, followed by generalized log transformation and data scaling by autoscaling (mean-centered and divided by the standard deviation of each variable) as described previously [35]. Chromatograms of metabolite measurements on various days for each type of product are shown in Supplementary Materials File S2, Figures S2–S10.

### 2.5. Data Analysis

Reported values are the average of two replicates obtained for every sample used for free amino acid, glycome, and lipidome analyses, and three replicates were used to obtain the average number of relative concentrations reported for peptidome data.

Peptide identification was performed using the Proteomicslfq version 1.0.0. software pipeline [36] (https://github.com/nf-core/proteomicslfq/tree/1.0.0, accessed on 12 December 2022) with the following parameters: input *. raw, --database *.fasta, --add_decoys, --protein_level_fdr_cutoff 0.1, --max_precursor_charge 7, --enzyme ‘unspecific cleavage’, --fixed_mods ‘ ‘--search_engines comet. Cow milk protein sequences from the UniProt database [37] milk_and_bovine_with_human.fasta were used, and the search engine was Comet [38]: https://uwpr.github.io/Comet/, accessed on 21 June 2023. Two bioactive peptide aggregator databases, BioPepDB [39] and MBPDB [40], were used to identify peptide function. Human proteins were included in the initial screening to exclude potential cross-contamination of peptides of human origin. An FDR threshold of 10% was selected for protein/group level, as it was more sensitive for the discovery-oriented analysis and allowed the recovery of candidate milk-derived peptide sequences. The fermented milk samples were analyzed without enzymatic digestion prior to LC/MS; therefore, an unspecific-cleavage search was chosen to avoid imposing tryptic cleavage constraints and to maximize the recovery occurring in peptide sequences for subsequent bioactive peptide annotation.

Data calculations and visualizations were conducted using R v4.3.3. Heatmaps were generated with a heatmap library. The color of the cells indicates the representation of each metabolite after logarithmic transformation and centering with respect to the mean value across all samples, where it was detected using the following formula: clr(x_i) = ln(x_i) − - mean(ln(x_1), … ln(x_n)), where x_i is the measurement of the compound in the i-sample; grey cells indicate levels below detection limits for measured compounds. Clustering of the metabolites and samples was also performed on normalized values, but centering was performed on all samples, including those where the metabolite was absent, by replacing unknown values with a small number. A cladogram for clustering the samples with important peptides was drawn using the hclust function. Bar plots were generated using the ggplot2 v4.0.2 and MicrobeR v0.3 packages. The correlation matrix was obtained with the corrplot v0.95 package, Spearman’s correlation and *p*-values after Holm’s correction for multiple comparisons were estimated with the rcorr.adjust() function from the RcmdrMisc v. 2.9-2 package. The Venn diagram was created with the ggVennDiagram v.1.4.8 package.

## 3. Results

### 3.1. Peptidome Characteristics

Analysis of peptidomes in the 15 products revealed 348 peptides (see Table S2). It is evident from the list of peptide representation in dairy products that fermented milk products contained more peptides than milk. Moreover, the peptide composition of 15 products was 75% similar, and peptides that were not common to all products were represented at approximately 10 times lower concentrations. When comparing the diversity of peptides in fermented milk products during storage, a slight change was observed; however, this was within technical variation of the method.

**Figure 2:**
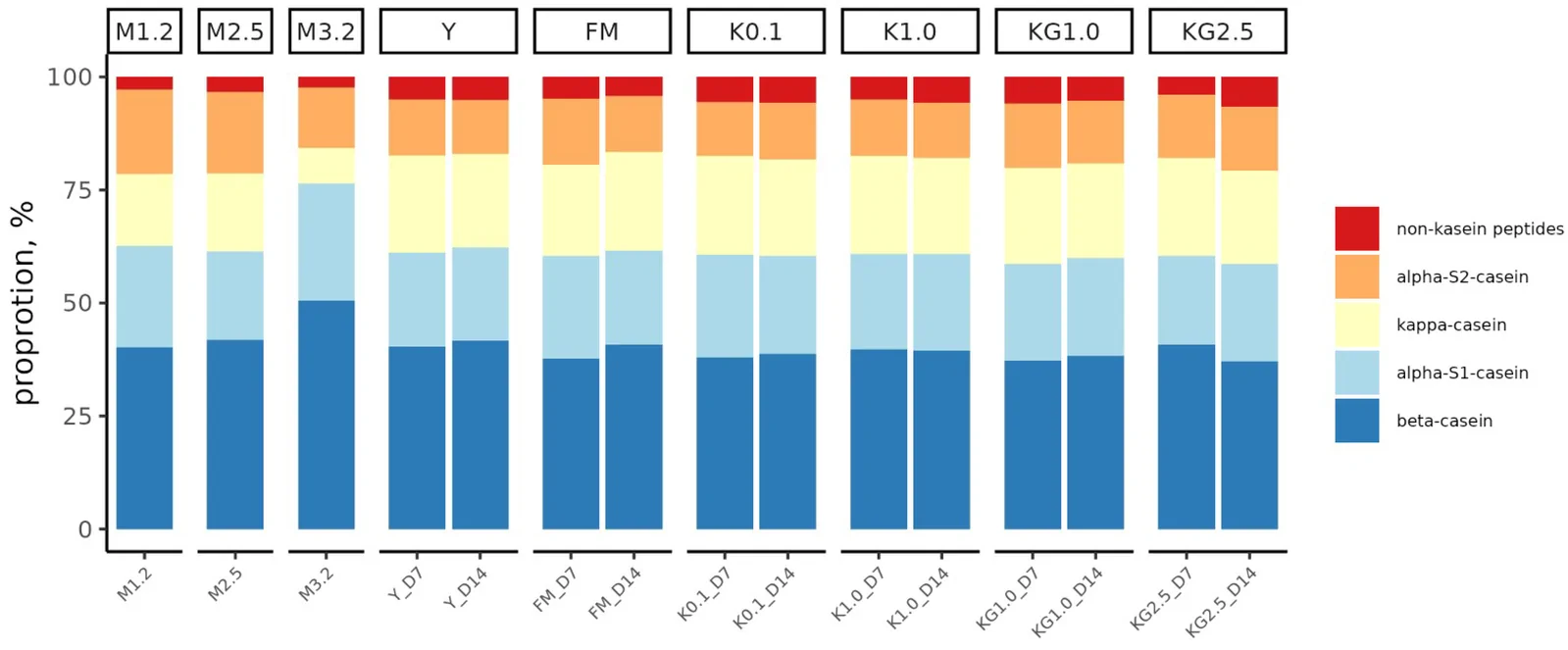
The ratio of the number of peptides derived from various protein fractions.

The predominant proportion of peptides observed in dairy products (94% on average) originates from the degradation of four proteins: 129 peptides from beta-casein, 72 peptides from kappa-casein, 75 peptides from alpha-S1-casein and 49 peptides from alpha-S2-casein (Figure 2, Table S2). The remaining peptides were represented by a few peptides and in negligible concentrations relative to the most represented peptides, which might be degradation products of casein proteins and originate from the following proteins of non-casein fractions: osteopontin (OSTP), perilipin-2 (PLIN2), butyrophilin 1/1A (BT1A1) and the glycosylation-dependent cell adhesion molecule 1 (GLYCAM1) (see Table S2).

Based on our study of dairy products, 37.4% of all peptides identified were products of the breakdown of beta-casein, 20.9% of kappa-casein, 21.7% of alpha-S1-casein and 20% of alpha-S2-casein.

When we examined the peptides based on their length and analyzed their accumulation during storage, they tended to form clusters according to their size (Figures S11–S14). In addition, there was a noticeable increase in the number of smaller peptides over time. This suggests that proteolysis during storage may lead to the generation of shorter peptides by breaking down larger peptides or proteins. The formation of peptide clusters and the increase in the number of smaller peptides indicate ongoing proteolytic activity and the dynamic nature of peptide composition during the storage of fermented milk products. It is possible that proteolysis might be localized at various sites of the original protein and the precursors of the target peptide and stop at the point when peptides form an inaccessible steady state for bacterial protease cleavage. In most cases, these peptides were functional based on matches with sequences from bioactive peptide databases.

**Figure 3:**
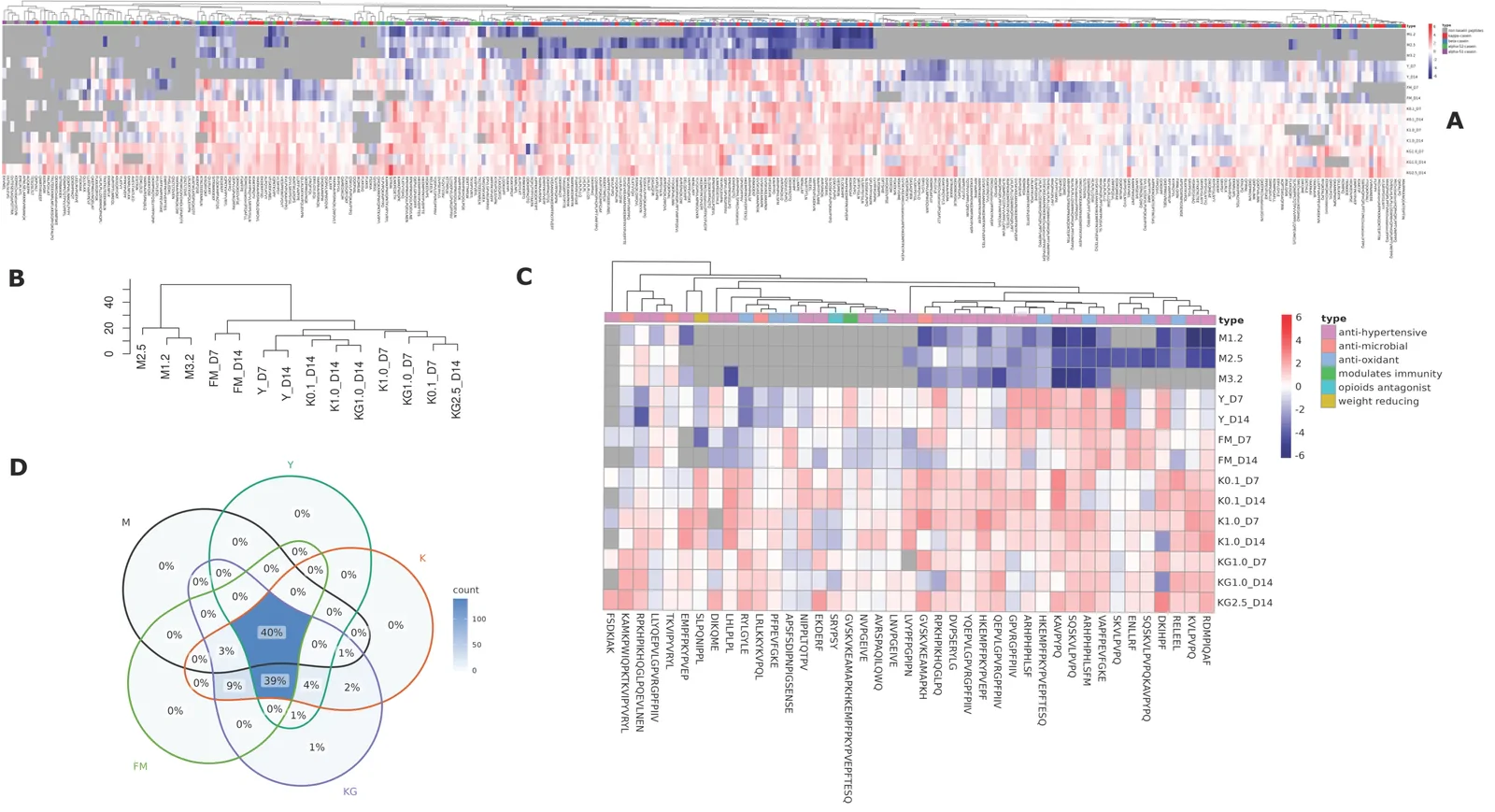
Profiles of 348 peptides in 14 dairy products. KG2.5_D7 was excluded from peptide comparison with other samples as it was analyzed in a separate batch. (**A**) Heatmap showing normalized concentration for all peptides identified by untargeted LC/MS; the fractions of milk proteins are reflected in the panel and colored. (**B**) Cluster dendrogram of fermented milk products and milk based on the peptide profiles. (**C**) Part of the heatmap representing only peptides with known physiological functions in humans. Fermented products are distinct from raw milk. Only peptides identified in fermented products have specific functions, such as modulating immunity and stimulating weight reduction. (**D**) Venn diagram showing the peptides shared between products and milk. Kefir with grains (KG) harbors 1% distinctive peptides compared to other products. There are three unique peptides that were identified with a 1% difference: KEPMIGVN, QEKNMAINPSKE and EPELPLHTL.

To assess the similarity of peptide composition profiles in fermented milk products, pairwise correlations of samples were performed using peptide composition. A clustering analysis was conducted based on the complete peptide profile (Figure 3B). Clustering was based on the type of product and resulted in three distinct clusters: M, Y and FM. For the initial time and samples taken after storage, each type of product was more distinct from the other categories than within the same product. This was not observed for kefirs K and KG, and their peptidomes did not change significantly during storage. Among the fermented products, FM and Y had the most distinct peptidome profiles. Although Y and FM demonstrated a peptide composition more similar to that of M than K and KG, the level of similarity remained relatively low.

This division into clusters may be attributed to the specific characteristics of fermentation and composition of the starter culture. Further interpretation of peptides of the fermented products versus raw milk revealed distinct specific peptide clustering (Figure 3A) within all analyzed peptides and also identified functional peptides with specific properties that were not found in milk (Figure 3C). We identified 41 functional peptides from the overall number of peptides reported (see Table S3). Notably, the number of functional peptides in milk was considerably lower than that in any fermented product, and products within the same time points were highly similar. Milk peptides appeared to contain low amounts of antihypertensive, antimicrobial, and antioxidant peptides, whereas KG showed the highest enrichment of additional functional peptides responsible for immunity modulation, weight reduction and opioid antagonists. Of note, KG had the highest overall diversity of peptides, and 1% of them did not appear in the other products analyzed in this study (Figure 3D). These included three unique peptides with unknown biological functions: KEPMIGVN, EPELPLHTL and QEKNMAINPSKE.

Most bioactive peptides appear to be unique, but some peptides have been reported previously. We identified the peptide YQEPVLGPVRGPFPIIV in our kefirs (K and KG), which not only exhibited antihypertensive activity but also antithrombotic, immunomodulatory, antioxidative and antimicrobial functions [41]. This peptide was also found in fermented milk with a single starter culture *Bifidobacterium longum* KACC91563 [42] and was produced by a mixed culture of *Streptococcus thermophilus*, *Lb. bulgaricus*, *Lb. acidophilus, Lb. casei* and *Lb. paracasei* [43]. Another identified peptide, HKEMPFPKYPVEPF, with antihypertensive functions in all fermented products, was previously reported to be a result of proteolysis by mixed cultures of *Lb. acidophilus*, *Lb. delbrueckii* subsp. *bulgaricus* and *Streptococcus thermophilus* [44].

We discovered enrichment of the functional peptide DKIHPF with angiotensin inhibitory properties in all fermented products. Previously, this peptide was reported only in goat milk and appeared to be eight times more active after pepsin digestion [45]. Peptides GVSKVKEAMAPKHKEMPFPKYPVEPFTESQ and SRYPSY have been previously found to function as agonists and antagonists, respectively, to opioid receptors in the gut, resulting in mucin production and an impact on peristaltic movement [46,47]. A single identified SLPQNIPPL peptide was previously reported to inhibit the activity of dipeptidyl peptidase 4, which is responsible for GLP-1 degradation in the cytosol [48].

Interestingly, the occurrence of all oligopeptides analyzed was not related to their origin from a specific protein or associated with their specific functions. This observation may be attributed to variations in the composition of proteases produced by bacteria and yeast present in the starter culture.

### 3.2. Free Amino Acid Profiles

**Figure 4:**
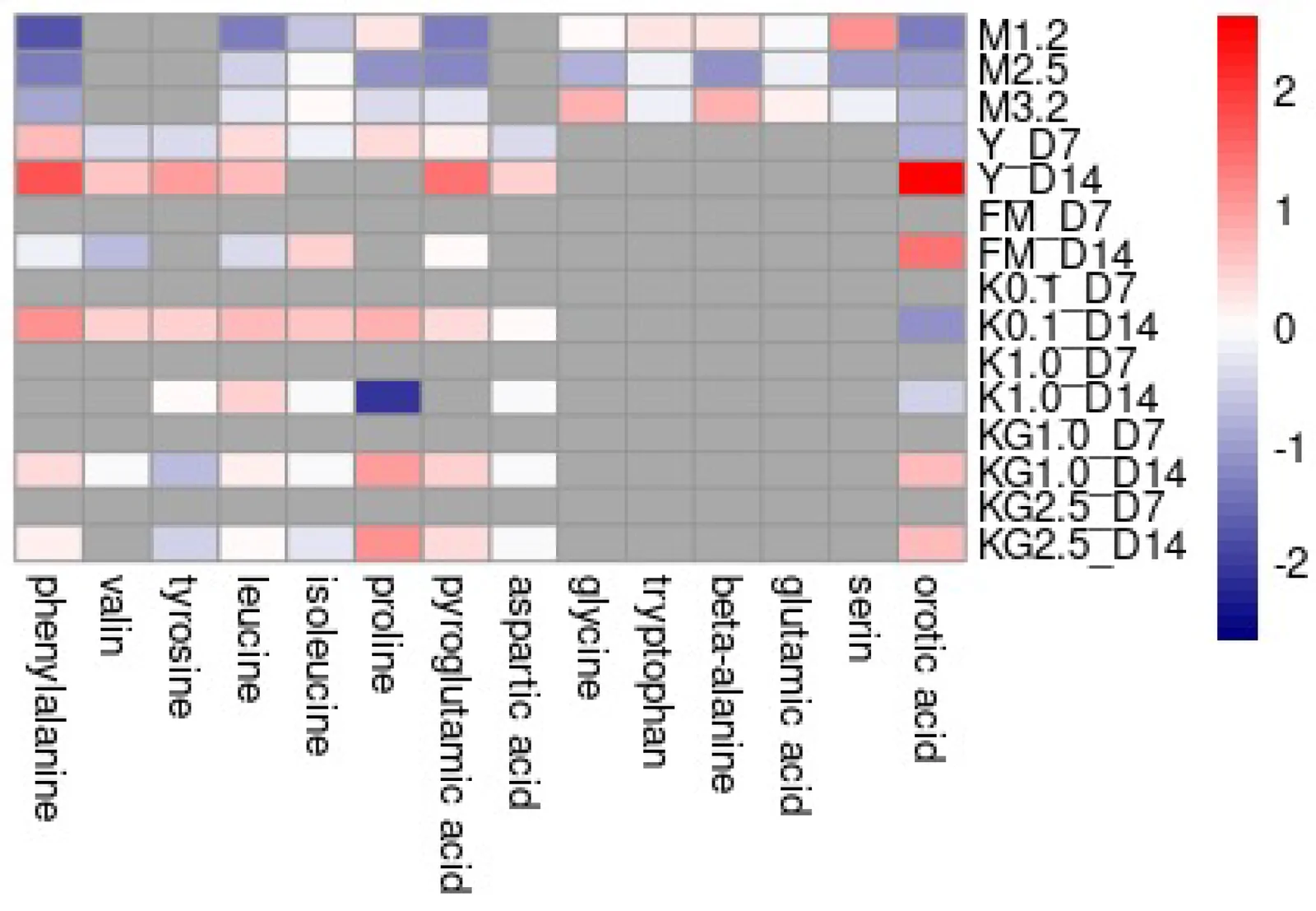
Heatmap of free amino acid and orotic acid concentrations in fermented products and milk.

The free amino acids were assessed using untargeted metabolomics by evaluating the obtained peaks relative to the library values. A search was conducted for 37 standard compounds, including the majority of biologically significant amino acids, including dipeptides (see Table S4). The sensitivity of this metabolomic method was quite low; therefore, only the most abundant analytes were detected. Figure 4 shows that the composition of free amino acids and orotic acid (vitamin B13) differed between milk and fermented products.

Moreover, there was a noticeable trend towards an increase in the amount of free amino acids, such as branched-chain amino acids (valine, leucine and isoleucine), during storage at D14, despite suboptimal conditions for proteolysis. Orotic acid accumulation was also observed at D14, whereas its known precursor molecules, such as aspartic acid and glutamic acid, were depleted. We also detected the accumulation of aromatic amino acids, such as phenylalanine and tyrosine, at D14, with the highest concentrations detected in Y compared with other fermented products. The common amino acids serine, alanine, glycine and tryptophan were not observed in any fermented product even at D7, although they were initially found in milk. This only shows the dynamics of certain amino acids by an active microbial community during cold storage. However, evaluation of absolute values is required for a more complete assessment of the amino acid profile using targeted metabolomic methods in future experiments.

### 3.3. Lipidome Analysis

**Figure 5:**
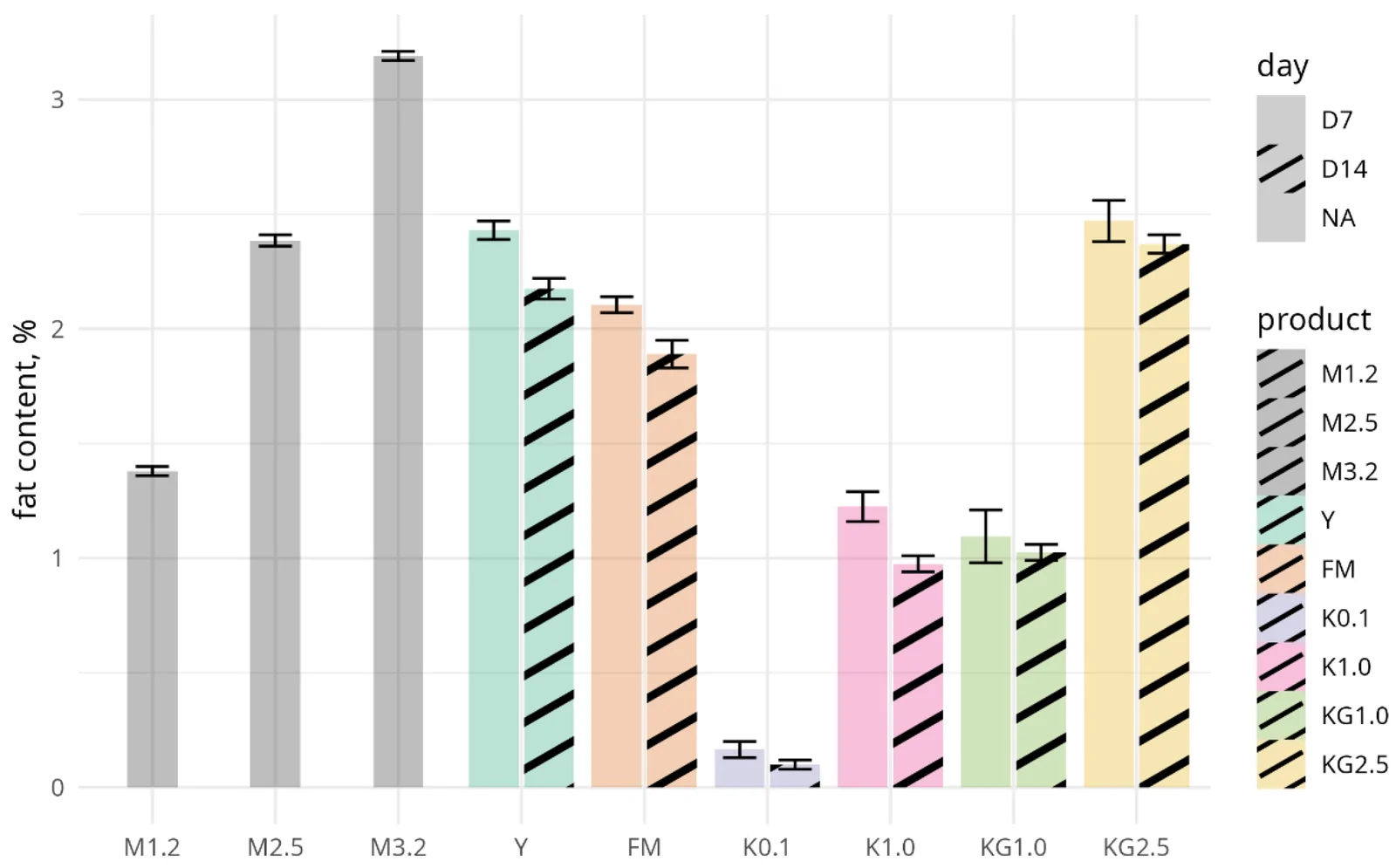
Triglyceride concentration with standard deviations in milk products based on fat content (see Table S7). Milk is shown in grey, fermented products are colored; dashed columns distinguish products stored for 14 days (D14) vs. 7 days of storage (D7).

We observed a decrease in the overall number of triglycerides present during the storage of fermented milk products (Figure 5). The fat and free fatty acid profiles of all fermented milk products were compared on days 7 and 14 of storage, revealing two distinct trends.

The table (Table S5) lists the fatty acids detected. Many of these fatty acids exhibit biological activity and have positive effects on the human body and microbiome. Among the identified fatty acids, polyunsaturated fatty acids, such as omega-6 and omega-9, were also identified. In addition to fatty acids, monoglycerides, which are likely products of triglyceride lipolysis, were also identified.

**Figure 6:**
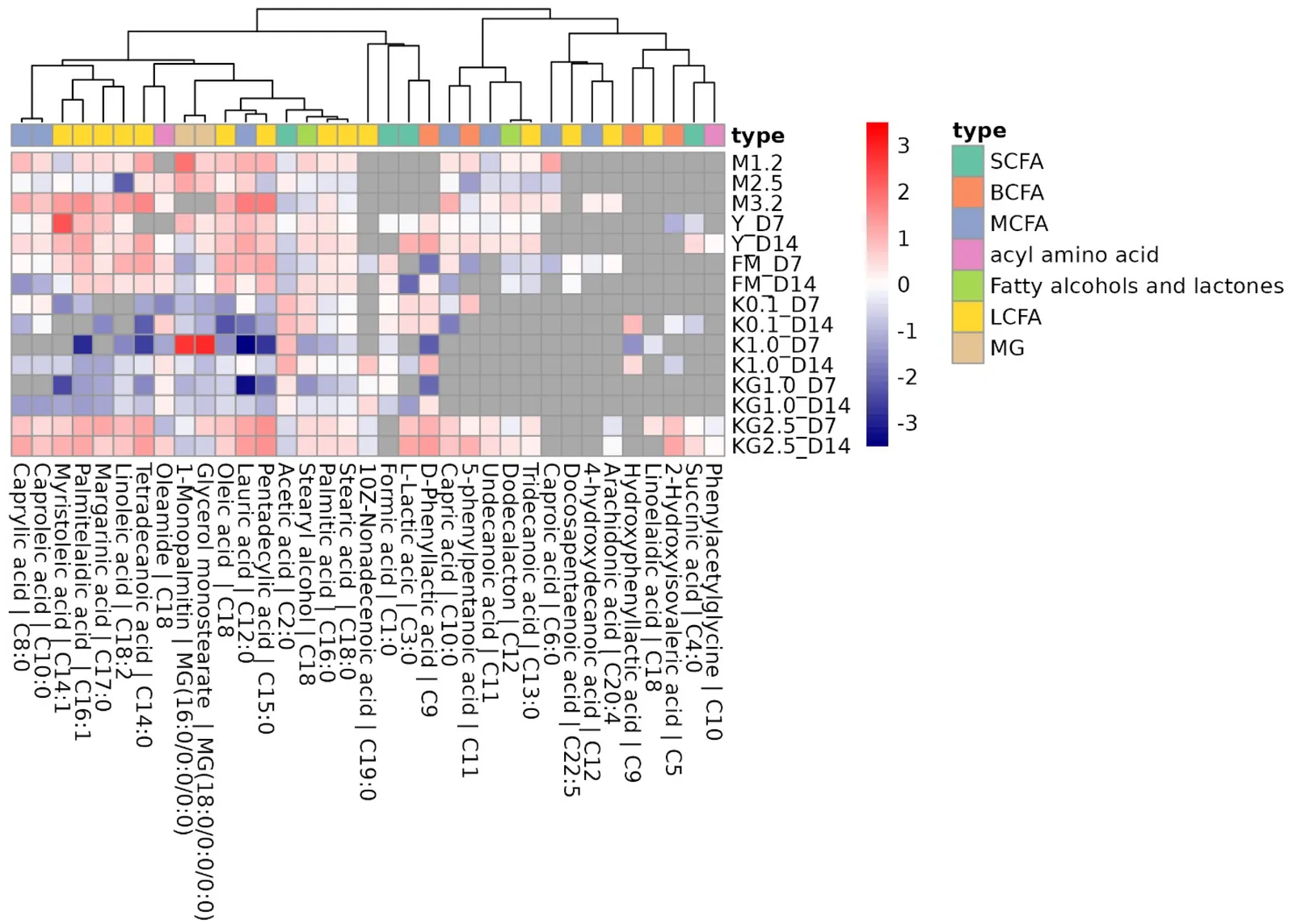
Heatmap of fatty acid concentrations in the products. Succinic acid was detected during fermentation in Y, K and KG but not in FM. Eleven metabolites that were lacking or measured only once are not shown on the graph: eicosapentaenoic acid, nonanoic acid, glutaric acid, dodecyl butyrate, 2-hydroxyisocaproic acid, butanoic acid, propionic acid, malonic acid, nonalactone, 5Z-dodecenoic acid, and dihomo-gamma-linolenic acid.

A comparison of the resulting clusters based on the representation of all identified compounds is displayed in the figure below as a heatmap (Figure 6). Notably, products with a fat content of over 1% exhibit distinctive characteristics of higher medium-chain fatty acid (MCFA) and long-chain fatty acid (LCFA) content.

When comparing fermented products with 2.5% fat content with products with 2.5% milk, it was observed that the quantities of medium- and long-chain fatty acids in products with 2.5% fat content were higher than those in milk with the same fat content (Figure S15).

Notably, the levels of monoglycerides and triglycerides in almost all products were lower than those in milk. This may serve as evidence of lipolysis during fermentation. A distinguishing characteristic of fermented foods is the presence of short-chain fatty acids, which were found at higher concentrations than in milk in all fermented milk products.

For product K with a single yeast and with 0.1% fat and for FM without yeast and with 2.5% fat, there was a noticeable decrease in triglyceride content, an increase in monoglycerides and a decrease in free fatty acids. In contrast, for K with 1% fat, Y with 1.7%, KG with 1%, and KG containing a mix of yeasts and with 2.5% fat, there was a decrease in total fat and monoglycerides, accompanied by an increase in long- and medium-chain free fatty acids compared to the other products (see Figure S16). This observation was likely due to the activity of different sets of yeast and bacterial lipolytic enzymes.

**Figure 7:**
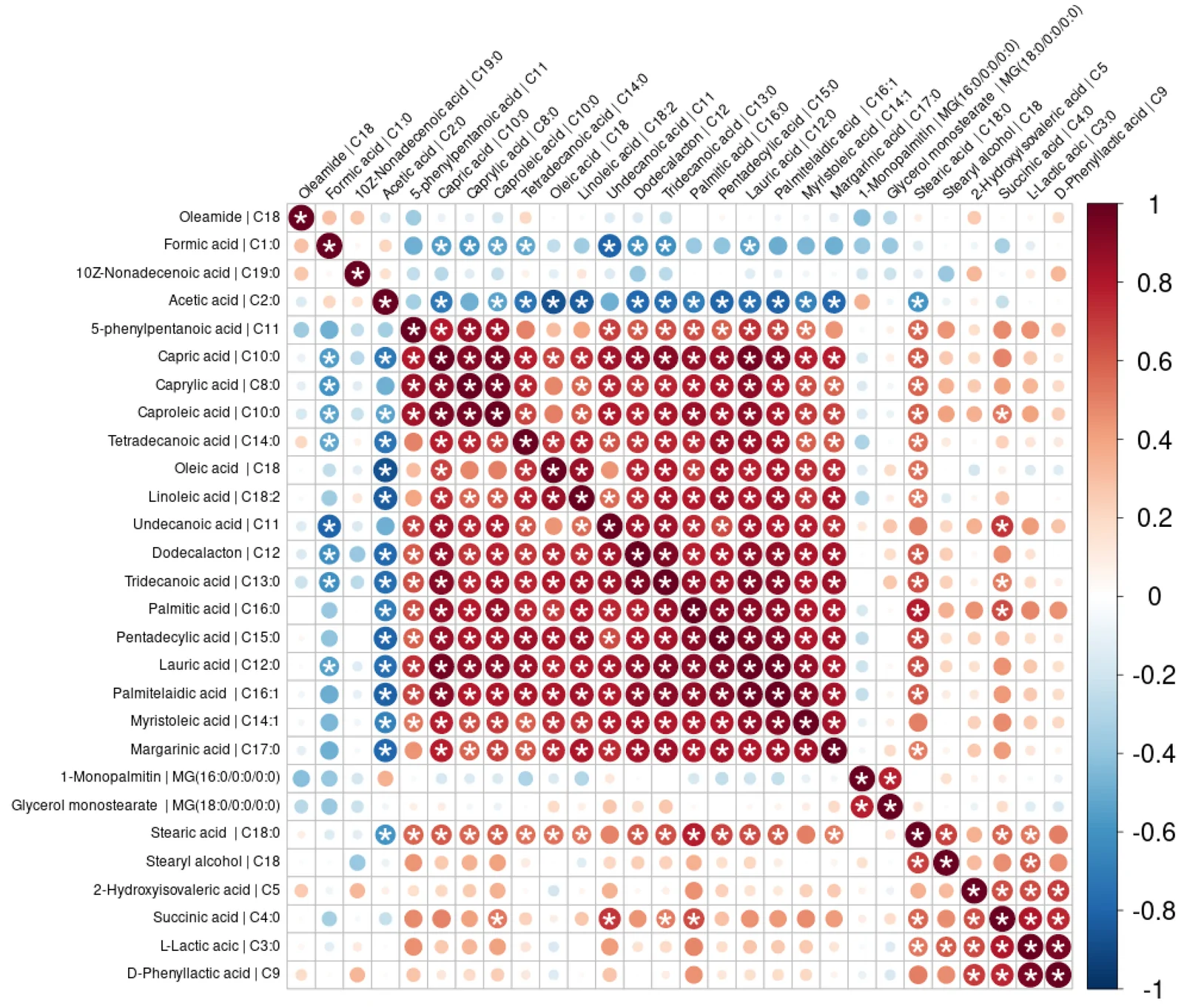
Correlation analysis of fatty acid composition. The values of each metabolite were compared for significance using Spearman’s correlation coefficient. Negative correlations are shown in blue, and positive correlations are shown in red. The diameter of the circles on the heatmap represents larger value correlations, and *p*-values after Holm’s correction for multiple comparisons > 0.05 are marked with asterisks.

There were more than 500 peaks after the library search, and only 37 compounds with a majority of fatty acids were identified (see Table S5). Correlation analysis of all identified compounds was performed to assess their possible co-occurrence. It can be seen that MCFA and LCFA were grouped into one large cluster, which can be explained by lipolytic processes of the decomposition of long chains into shorter ones, as well as the synthesis of protective antimicrobial compounds (Figure 7). A negative correlation was observed between formic acid and MCFA (e.g., caprylic acid) and LCFA (e.g., undecanoic acid). In contrast, a positive correlation was observed between succinic acid and palmitic acid. Another cluster was formed due to positive correlations between SCFA and BCFA, such as L-lactic acid, β-phenyl lactic acid, 2-hydroxy-valeric acid, and saturated stearic acid (C18:0). Monoglyceride 1-monopalmitin was positively correlated with monoglyceride glycerol-stearate, which may be due to specific microbial lipase activity. To interpret the biological significance of these correlations, a more in-depth study of the metabolic pathways of the bacteria and yeast present in foods is necessary.

### 3.4. Glycome Analysis

The profiles of mono- and disaccharides and polyol myo-inositol were determined using untargeted metabolomics analysis. More than 500 peaks were identified per sample for each product. These peaks were then compared with the library values for simple sugars, resulting in the assessment of values corresponding to 23 standards (Table S6).

**Figure 8:**
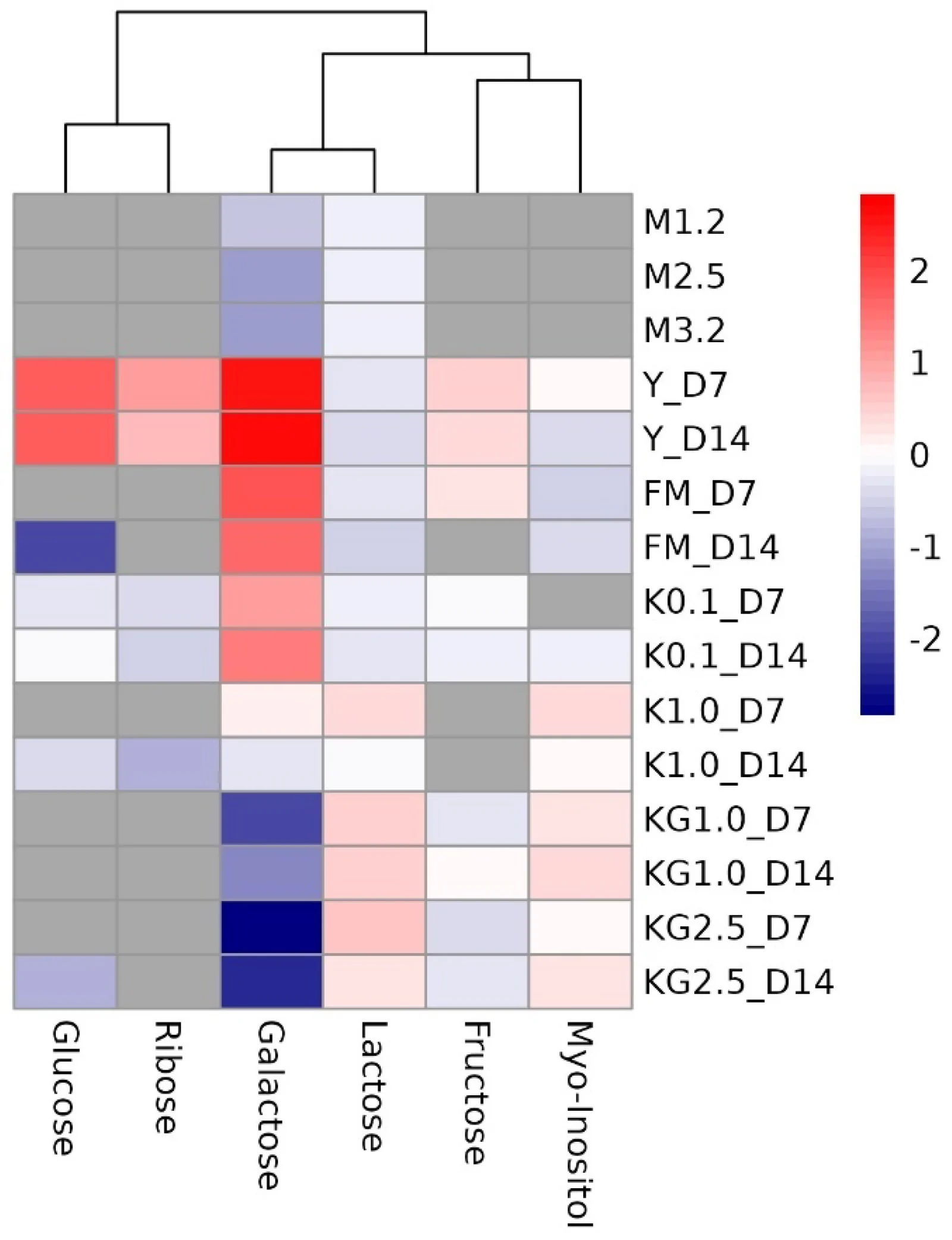
Heatmap showing changes in the profile of mono- and disaccharides in fermented milk products during storage (7 vs. 14 days).

The decomposition products lactose, glucose and galactose were present in the fermented products (Figure 8). This indicates active lactose decomposition and further metabolism of monomers during storage. Importantly, no accumulation of simple sugars was observed, suggesting that these compounds were actively utilized in other bacterial processes. Overall, the analysis of simple sugar profiles using untargeted metabolomics provides insights into the decomposition and utilization of lactose in fermented milk products. Furthermore, during the life processes of microorganisms, other sugars such as fructose and ribose may also be produced. In addition to sugars, the presence of myo-inositol was identified. In addition to comparing the composition with milk, changes in the levels of simple sugars during storage were evaluated.

Lactose levels were noticeably higher in K and KG than in Y and FM. At the same time, the increase in the amount of different simple sugars occurred differently for all products, probably because of the composition and metabolic potential of the fermenting microbial community. In contrast, an increase in the galactose content was observed in Y, FM, and K with 0.1% fat, indicating active lactose fermentation by the microbial community during storage.

We observed an accumulation of myo-inositol, especially at D14, in all fermented products. This polyol has many protective functions for health and was not detected in the milk samples.

## 4. Discussion

This study was based on the untargeted peptidome and metabolome profiling of four types of fermented dairy products and revealed 348 varieties of peptides, 27 fatty acid compounds, and the accumulation of BCAAs (valine, leucine, isoleucine), orotic acid and myo-inositol after 14 days of cold storage, resulting from metabolic activity during milk fermentation. The number of peptides and fatty acids decreased slightly, but not significantly, on day 14, in contrast to that on day 7 of storage. We found that 1% of the total peptides identified were unique to KG compared to Y, FM and K, made with commercial starter cultures. These specific peptides might be the product of the diverse symbiotic microbial community of the wild kefir grains of KG, which includes more than 10 species of yeast, compared to K with a single yeast present (see Table S8). One of the dominant yeast species found in KG was *Malassezia* spp., which accounted for 26% of the total fungal abundance. This yeast may be responsible for the specific metabolite profile, as it is not usually found in kefirs, which are well characterized [49], but is reported to be the primary yeast responsible for the fermentation of kombucha in Brazil [50]. Since this yeast was identified in kefir grains for the first time, experimental evidence of the metabolic role in taste formation of fermented dairy products will be required.

The majority of peptides (94%) originated from casein degradation. This is not surprising because the primary source of these peptides is casein, which constitutes up to 80% of the milk protein fraction [51]. The casein family consists of four main types: alpha S1-casein, alpha S2-casein, beta-casein, and kappa-casein. These caseins are widely present in various food products, ranging from being the primary constituent of cheese to serving as food additives. Although all four casein proteins are related, multiple alignments of their amino acid sequences using the CLUSTAL-Omega v1.2.4 program [52] indicated minimal homology between the sequences.

Peptides from other proteins were present in smaller quantities, whereas we did not detect peptides from the bovine whey fraction, including α-lactalbumin (α-LG), β-lactoglobulin (β-LG), lactoferrin and lactoperoxidase, which account for 70–80% of the total whey fraction. Similar results have been reported for other starter cultures, which can be explained by poor proteolysis during fermentation [53]. In contrast, later studies on kefir from goat milk identified four α-LA peptides and 14 β-LG peptides after 36 h of storage, and none of the α-LA and two β-LG peptides were found in raw goat milk [54]. In the current study, we analyzed fermented cow dairy products after seven days of storage and did not find any lactalbumin peptides. Their lack might be explained by the following: (i) few peptides might degrade during prolonged days of storage; (ii) the lactalbumin fraction was initially present in cow’s milk in very small numbers compared to goat’s milk; (iii) lactalbumins are more resistant to the microbial peptidase present in our commercial products, which was also reported previously [55], and therefore might be missed by the LC/MS protocol focused on the detection of relatively short peptides.

Based on our study, it appears that most peptides resulting from the proteolysis of beta-casein in fermented milk products were not found in raw milk. Additionally, the peptides present in milk were observed at minimal concentrations. This suggests that proteolysis during the fermentation of milk products generates unique peptides that are not commonly found in milk. In this study, milk was used as a reference only for comparison with other products, as it would not be feasible to use milk as the initial source for every corresponding commercial fermented product. The technological chain of a particular fermented dairy product involves a mixed formulation consisting of local normalized milk, skim milk powder, and the addition of whey, if necessary, to adjust the protein and fat contents. Hence, we chose locally normalized milk with a range of fat contents. Furthermore, the composition of metabolites in milk can vary depending on various factors, such as the conditions under which cows are maintained and seasonal variation. Therefore, we admit that this difference in milk used as a reference may have introduced some variability in the results obtained during the study.

These findings emphasize the significant impact of microbial proteolysis on the composition and characteristics of fermented milk products. In general, kefir had the largest pool of peptides compared to the yogurt and fermented milk analyzed in this study, which is in agreement with previous studies [56].

In this study, 41 functional peptides were identified. Common examples of very short peptides absorbed by the intestine that have known physiological activity in the blood are valine-proline-proline (VPP) and isoleucine-proline-proline (IPP). Such peptides are formed from milk protein as a result of the protease activity of *Lactobacillus helveticus* and other LAB. However, we did not detect these short functional peptides despite the presence of dipeptide peaks below the resolution levels (Table S2). Some long peptides with a size of 10–51 amino acids (e.g., gonadotropin-releasing hormone 1 and insulin) remain intact, can be absorbed in the intestine, and may have physiological effects on cells. All functional peptides identified in this study belong to the relatively long peptides.

Of note, during fermentation at D14, we observed the accumulation of branched chain amino acids (valine, leucine, iso-leucine), phenylalanine, tyrosine, proline, aspartate, pyroglutamic acid and orotic acid (vitamin B13), whereas tryptophan, glycine, beta-alanine, serine and glutamate were consumed on D7 of the product. Thus, the proteolytic activity of the microbial community may lead to the accumulation of certain amino acids from casein [57], and BCAAs from Greek-style yogurt may elicit different postprandial aminoacidemic responses [58]. Accumulation of orotate in KG and FM, with the highest concentrations in Y, was observed on D7 and D14, and our results support a single study [59] suggesting that microbial synthesis of vitamin B13 might occur under refrigerated conditions despite an initial loss reported for milk under simulated fermentation [60].

The composition of the fat fraction, which consists of triglycerides and free fatty acids, was highly dynamic. Longer compounds, such as tri- and monoglycerides, were broken down into free fatty acids. Fatty acids may be broken down into shorter fatty acids; however, the medium-length fatty acid fraction did not accumulate significantly, suggesting possible microbial utilization. The short-chain fatty acid fraction was the most highly represented in all fermented products, with succinic acid being the most abundant in KG. Lactobacilli, present at high levels in dairy products, are known for their ability to produce lactate and acetate, and the removal of the bacterial community from the reconstituted kefir community may lead to the persistence of other secondary metabolites [61], including succinic acid, which is abundant in carbohydrates [62]. Yeasts can form succinic acid from glutamate, as shown in the C13-labelled substrate experiment [63], and we observed the utilization of glutamate in KG. In yogurt, *L. bulgaricus* plays a primary role in succinic acid production from fumaric or malic acids, as demonstrated in the reductive branch of the TCA cycle [64].

Kefir consumption was shown to alleviate autism-like behavior in a mouse model of ASD and increase anti-inflammatory Treg cells in the lymph, with increasing succinic acid, elevated *Lachnospiraceae* bacterium A2 taxa, and decreased *Clostridiaceae* abundance in the gut [65]. Recently, *Lachnospiraceae* A2 taxon was found to drive IgA levels in the intestine [66]. Thus, these positive effects of succinic acid might be translated to humans but must be considered with caution. Strains of *L. kefiranofaciens,* which are also found in kefir grains of KG but are present in low abundance (0.1%) in the final product, involve upregulation of Treg cells [67], consequently inhibiting the secretion of proinflammatory markers but also inducing obesity in high-fat diet (HFD)-fed mice [68].

Some functional metabolites, including propionic, maleic, butyric, dihomo-gamma linoleic (DGLA) and eicosapentaenoic acid (EPA), were not observed, which may indicate their very low concentrations in the analyzed samples. We also did not detect a gut barrier-protective conjugated linoleic acid (CLA), with very low concentrations of this microbial fatty acid, such as 0.06–0.13 mg/g, in the fermented products [69,70]. The introduction of specific starter cultures, such as *Lactococcus lactis* ssp. *cremoris* MRS 47, may increase CLA concentrations [71].

As for carbohydrate components, the fermentation process breaks down lactose into glucose and galactose, which is likely to have a favorable effect on the digestibility of products, especially for people with lactase deficiency, which, as we demonstrated earlier, tends to persist in up to 43% of East Slav genotypes [72]. Furthermore, simple sugars, such as glucose and fructose, were released during lactose fermentation, primarily in Y. These simple sugars may contribute to the overall taste of the products. Overall, glycolysis during fermentation leads to the breakdown of lactose into glucose and galactose, which aids in digestibility. This also results in the release of simple sugars and other compounds, contributing to the taste and potential health benefits of fermented products.

Additionally, other sugar-like compounds, such as myo-inositol, were released during fermentation. Myo-inositol is a component of phytate, which is common in cereals and legumes, but can also be released as an intermediate product of glucose metabolism by microorganisms [73], including the primary KG member *K. marxianus* [74]. To our knowledge, myo-inositol has been reported as an end product in yogurt made from sheep’s milk [75], originating from a raw source. In contrast to that study, we did not observe the source of myo-inositol in cow’s milk but observed accumulation of this compound in K on day 7 and in K on day 14 with fats >1%, while Y and FM had very low relative quantities. Myo-inositol has a wide range of biological activities, including participation in protection from type 2 diabetes via the reducing blood glucose concentration in metabolic disorders related with insulin resistance and regulation of central nervous system activity and can potentially serve as a prebiotic for butyrate-producing gut microbes [76,77]. Currently, myo-inositol is being tested as a treatment for disorders of the nervous and reproductive systems and diabetes, including gestational and malformation disorders [78]; this polyol was highlighted in a meta-review, where it was reported that it can reduce gestational diabetes mellitus [79].

In addition to the main components of the products, we found a number of low-molecular-weight compounds that are considered to play a role in body functionality. D-phenyl lactic acid, a product of phenylalanine metabolism by bacteria, exhibits antimicrobial activity. Thus, it can be used as an eco-friendly agent for food preservation [80]. It is also described as a bacterial metabolite capable of specifically modulating the immunity and energy system of the human body through activation of the HCA3 receptor [81]. The acylated amino acid oleamide may be formed as a result of the enzymatic degradation of phospholipids. It is an intermediate metabolite that participates in arachidonic acid synthesis catalyzed by fatty acid amide hydrolases. This compound has been actively studied as an endocannabinoid ligand to improve sleep quality and psychological health [82]. Orotic acid is involved in the biosynthesis of pyrimidines and proteins and is essential for the regulation of genes that play a role in the development of cells, tissues, and organisms. It also plays an antioxidant role and is associated with cardiac health (vasorelaxation and cardioprotection) [83]. The metabolic activity of biotics is the main factor affecting the health impact of consumed fermented products. Among all metabolite classes analyzed in the current study, bioactive peptides remain promising for further detailed studies.

There are several limitations that are important to note. First, the milk samples were not the same as those used to produce the fermented products. The technological chain of a particular fermented dairy product involves a mixed formulation consisting of locally normalized milk and skim milk powder, with the aim of adjusting key parameters, such as protein and fat, for a standardized fermentation process in the dairy plant and to maintain the nutritional values on the label of the product. The composition of metabolites in milk can also vary depending on various factors, such as the conditions under which cows are maintained. This difference in milk used as a reference may introduce some variability in the results obtained during the experiment. Second, the presence of a D0 sampling point would be helpful to estimate metabolites at early post-processing, in addition to D7 and D14. However, we must emphasize that it is not feasible to have fresh dairy products on supermarket shelves with a production date of D0 due to the strict quality assurance requirements of factories and the logistics delays of cold chain delivery. The results of the industrial production of a wide range of dairy products presented in this study include a strong rationale for comparing the results of D + 7, which is the most probable day that the product will appear on the shelf. Third, more biological replications are required to consider the variation in the product metabolite composition as a result of technological variation in production batches at dairy plants. Non-target or semi-quantitative proteomics methods were used to detect peptides in the sample, but only very rough conclusions could be drawn regarding the concentrations of the detected peptides, indicating whether one peptide was present at higher or lower concentrations than the other. To accurately estimate the absolute quantities of the compounds of interest, it is necessary to perform targeted analysis of sample product batches. Some of the 348 detected peptides were observed at very low concentrations. The number of peptides present at very low concentrations in the samples exceeded the sensitivity of the method. We also would like to emphasize an important limitation that concerns statistical analysis of the data. The data are compositional, that is, they describe only relative, not absolute, information and contain many zeros. Current compositional methods are not directly compatible with zero values; therefore, zero-replacement was applied. Despite being a common strategy, this may still introduce bias into the results, possibly giving too much weight to the presence or absence of features.

## 5. Conclusions

Milk fermentation driven by microbial activity significantly alters the composition of the product during a cold storage of 14 days. Proteolysis releases a diverse array of peptides, primarily casein, which tends to boost protein bioavailability and ceases at the stage of small non-degradable bioactive peptides. Lipolysis results in a shift towards shorter-chain fatty acids, likely due to the microbial utilization of medium-length chains. Glycolysis enhances lactose digestibility, releases simple sugars that affect the overall flavor of the product, and generates additional bioactive compounds. These transformations contribute to the unique nutritional and functional properties of fermented milk. However, variations in raw materials and processing conditions require targeted analysis to accurately determine the absolute quantities of beneficial compounds for each product type.

## Figures and Tables

**Table 1:** Microbial composition and fat content of products used in this study. To study the decomposition dynamics of these substances, two samples were collected on days 7 (D7) and 14 (D14). Only microbial species with > 1% abundance are shown for the KG samples.; n.a.—not applicable.

ID	Description	Starter Cultures		Fat, %	Sample ID
		Bacteria	Yeasts		
KG	Kefir made with grains	*Lactococcus lactis* *Stenotrophomonas maltophilia* *Leuconostoc mesenteroides* *Lactobacillus acidophilus* *Bifidobacterium animalis* *Lactobacillus kefiranofaciens*	*Kluyveromyces marxianus* *Kazachstania humatica* *Kazachstania unispora* *Malassezia restricta* *Malassezia* sp. M8 *Malassezia globose* *Saccharomyces cerevisiae* *Candida santamariae* *Athelia bombacina* *Salvia reuteriana*	1.0	KG1.0_D7 KG1.0_D14
				2.5	KG2.5_D7 KG2.5_D14
K	Kefir drink made with commercial cultures	*Lactobacillus acidophilus* *Lactococcus lactis* *Lactococcus lactis cremoris* *Lactococcus mesenteroides* *Bifidobacterium animalis* subsp. *lactis*	*Debaryomyces hansenii*	0.1	K0.1_D7
					K0.1_D14
				1.0	K1.0_D7
					K1.0_D14
FM	Fermented milk	*Streptococcus thermophilus* *Lactobacillus acidophilus*	n.a.	2.5	FM_D7
					FM_D14
Y	Probiotic yogurt	*Lactobacillus delbrueckii* subsp. *bulgaricus* *Streptococcus thermophilus* *Lactococcus lactis* *Bifidobacterium animalis* subsp. *lactis*	n.a.	1.7	Y_D7
					Y_D14
M	Milk	n.a.	n.a.	1.2	M1.2
				2.5	M2.5
				3.2	M3.2

## Data Availability

Data described in the manuscript are available without restriction at https://doi.org/10.5281/zenodo.15720835. The original contributions presented in this study are included in the article. Further inquiries can be directed to the corresponding author.

## Supplementary Materials

The following supporting information can be downloaded at: https://www.mdpi.com/article/10.3390/cimb48090955/s1.

## Abbreviations

ACN	acetonitrile
CAA	2-chloroacetamide
CLA	conjugated linoleic acid
DCNa	sodium deoxycholate
FM	fermented milk
GC-MS	gas chromatography–mass spectrometry
IPP	isoleucine-proline-proline
K	kefir drink
KG	kefir made with wild kefir grains
LAB	lactic acid bacteria
LCFA	long-chain fatty acid
M	normalized milk
MCFA	medium-chain fatty acid
MSTFA	N-Methyl-N-(trimethylsilyl)-trifluoroacetamide
TCEP	tris(2-carboxyethyl) phosphine
TFA	trifluoroacetic acid
UPLC-MS/MS	Ultra-performance liquid chromatography–mass spectrometry
VPP	valine-proline-proline
α-LA	alpha-lactalbumin
β-LG	beta-lactoglobulin
