# Supplementary material for: Peptidome and Metabolome Profiling of Fermented Milk Products Reveal Accumulation of Bioactive Compounds During Cold Storage
Source: Curr Issues Mol Biol. 2026 Sep 19;48(9):955. doi: 10.3390/cimb48090955 (PMC13605456; doi:10.3390/cimb48090955)
Supplement: Supplementary file 1 [file cimb-48-00955-s001.zip › Supplementary materials_File S1.pdf]

Supplementary Materials File S1

QC protocols for peptidome and metabolomics

1. **Calibration standards for peptides**

For each sequence, chemical synthesis of both the target KVLPVPQ and the modified peptide KVLPVPQA was performed by adding an additional amino acid as a non-isotopic tag. The peptides were chromatographically purified and their concentrations were spectrophotometrically measured by absorbance at 214 nm. The molar extinction coefficients of the synthetic peptides were determined using ProtPi (https://www.protpi.ch/Calculator/PeptideTool) with their corresponding amino acid sequences. Final concentrations of purified peptides KVLPVPQ and KVLPVPQA were 0.4 μg/μL and 0.7 μg/μL correspondently.

All synthetic peptides were mixed in a single solution. To construct the calibration curve, a calibration panel of 12 concentrations in 3.3-fold increments was prepared using resulting calibrator by serial dilution. Mass spectrometry analysis was performed on each of the 12 calibration panel samples. Raw data from the analysis of the control samples in *.raw format are available at: 10.5281/zenodo.14751156

For all synthetic peptides, plots of the dependence of peptide MS peak intensities on the actual peptide concentration in the sample were constructed. For all peptide pairs, linear dependence of the two parameters was demonstrated within the range of concentrations used (R-value $\geq$ 0.95) (see Figure S1).

2. Metabolomic standards and consistency of free fatty acids measurements on different dates.

Each metabolite peak area was normalized to the internal standard of 2-iso-propylmalic acid (Sigma-Aldrich, cat #333115) for carbohydrates and amino acids; Supelco37 standard (Sigma-Aldrich, cat #CRM47885) for free fatty acids followed by generalized log transformation and data scaling by autoscaling (mean-centered and divided by standard deviation of each variable) as described before [1].

Comparative analysis of the relative concentrations of FFAs in milk and fermented milk products. The chromatograms for each sample category are presented at Supplementary materials File S2 (see Figures S2-S10).

In milk samples with 1.5%, 2.5%, and 3.2% fat percentages taken at different dates, there were no changes in the relative concentrations of free fatty acids (see Figures S2-S4).

In Kefir with commercial yeast monoculture (K0.1 and K1.0), there were higher amounts of stearic acid and amide of oleic acid on day 14 (D14) than on day 7 (D7) of storage (see Figure S5-S6).

For the fermented milk (FM) samples, a slight increase in stearic acid content was observed (see Figure S7).

For kefir on grains with 1% fat (KG1.0), the appearance of new peaks corresponding to arachidonic, decanoic, and 5-phenyl valeric acids with slight decreases in oleic, stearic, and amide oleic acids were observed after 14 days of storage (Figure S8). In KG2.5, lactic acid, stearic acid, and oleic acid slightly increased during storage, and new peaks corresponding to arachidonic acid and 5-phenyl-valeric acids were observed (see Figure S9).

For yogurt samples (Y), there was a profound increase in the relative amounts of lactic, oleic, succinic, and stearic acids, and a slight increase in 5-phenyl valeric acid and glycerol monostearic acid at D14 compared to D7 (see Figure S10).

References:

1. Salzano, A.; Manganiello, G.; Neglia, G.; Vinale, F.; De Nicola, D.; D'Occhio, M.; Campanile, G. A Preliminary Study on Metabolome Profiles of Buffalo Milk and Corresponding Mozzarella Cheese: Safeguarding the Authenticity and Traceability of Protected Status Buffalo Dairy Products. *Molecules* **2020**, *25*, 304, doi:10.3390/molecules25020304.
