# Supplementary material for: Peptidome and Metabolome Profiling of Fermented Milk Products Reveal Accumulation of Bioactive Compounds During Cold Storage
Source: Curr Issues Mol Biol. 2026 Sep 19;48(9):955. doi: 10.3390/cimb48090955 (PMC13605456; doi:10.3390/cimb48090955)
Supplement: Supplementary file 1 [file cimb-48-00955-s001.zip › Supplementary materials_File S2.pdf]

Supplementary materials File S2

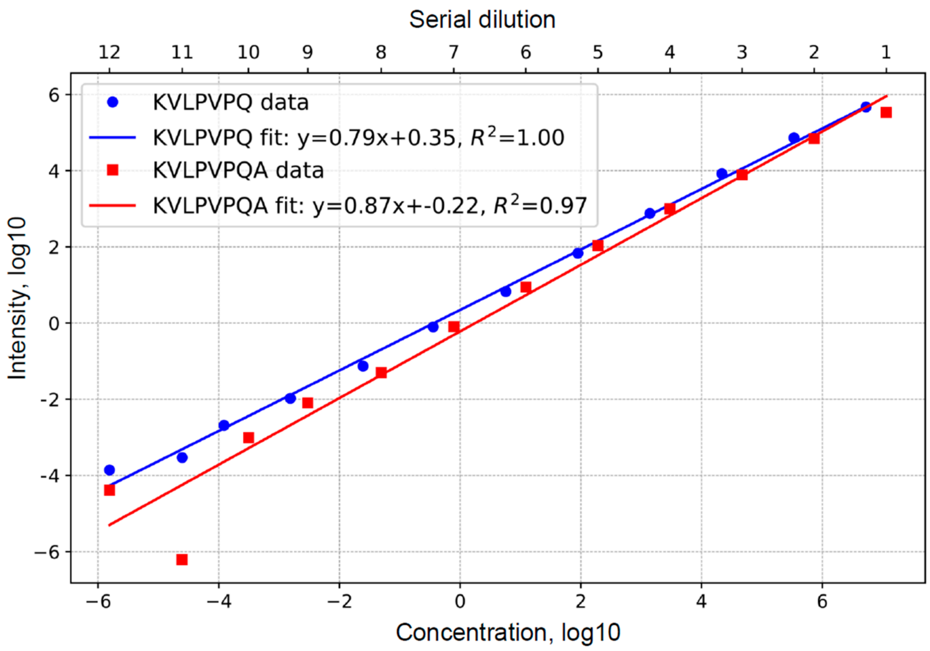

Figure S1. Calibration curve showing intensity vs concentrations of synthetic KVLPVPQ and KVLPVPQ(A) peptides standards

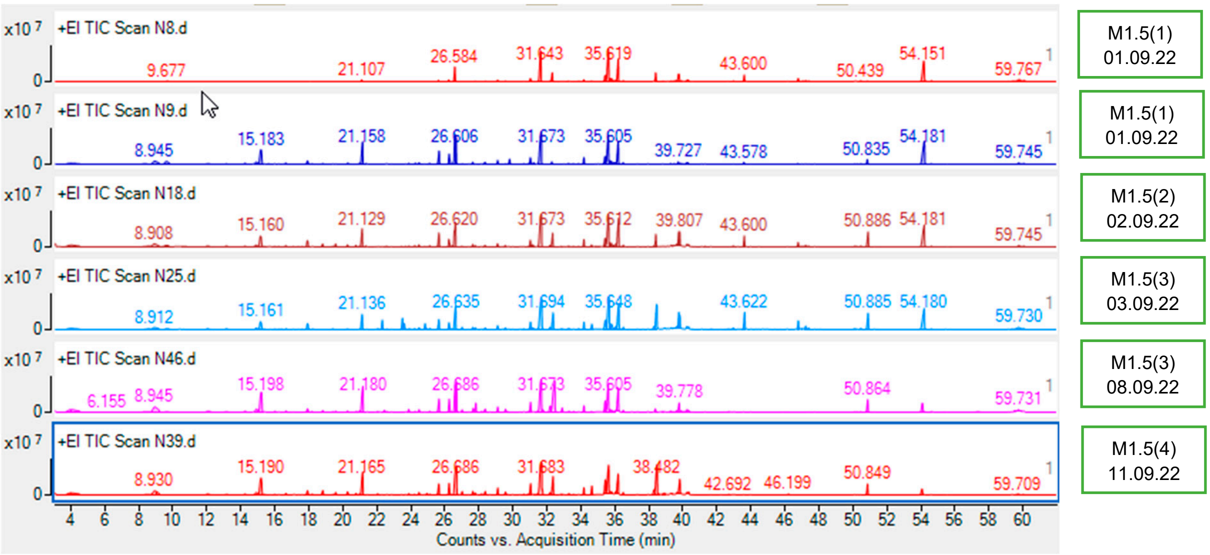

Figure S2. Milk samples with 1.5% fat (M1.5) chromatograms.

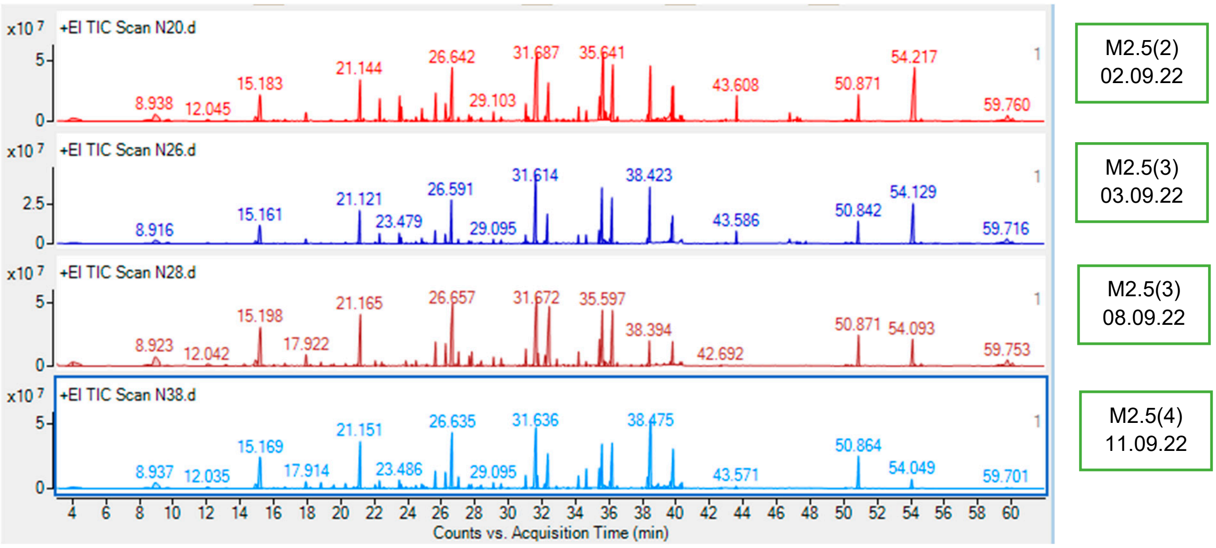

Figure S3. Milk samples with 2.5% fat (M2.5) chromatograms.

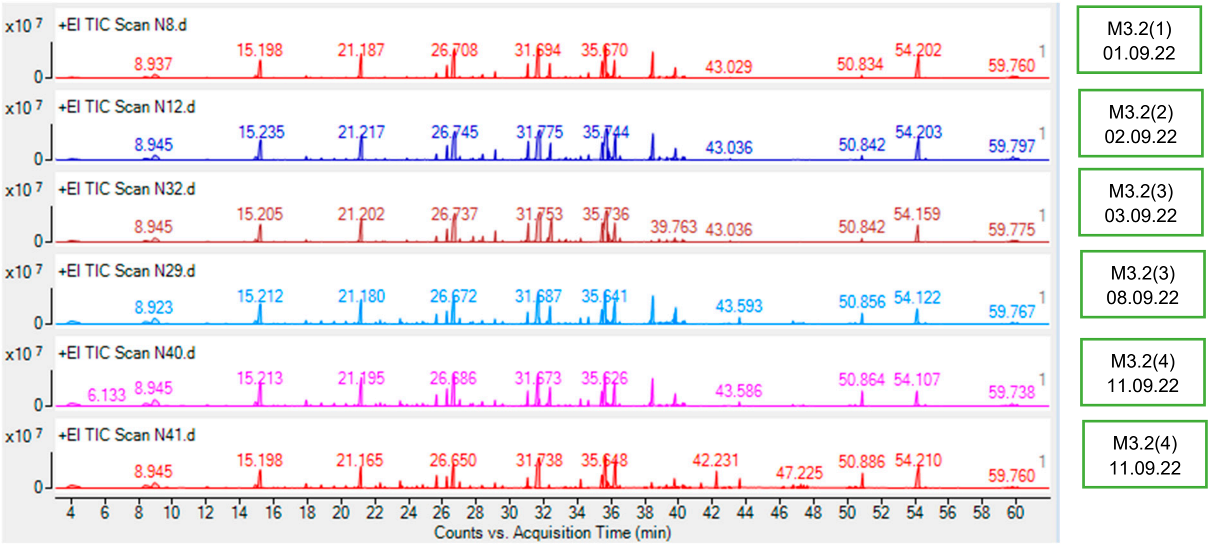

Figure S4. Milk samples with 3.2 % fat (M3.2) chromatograms.

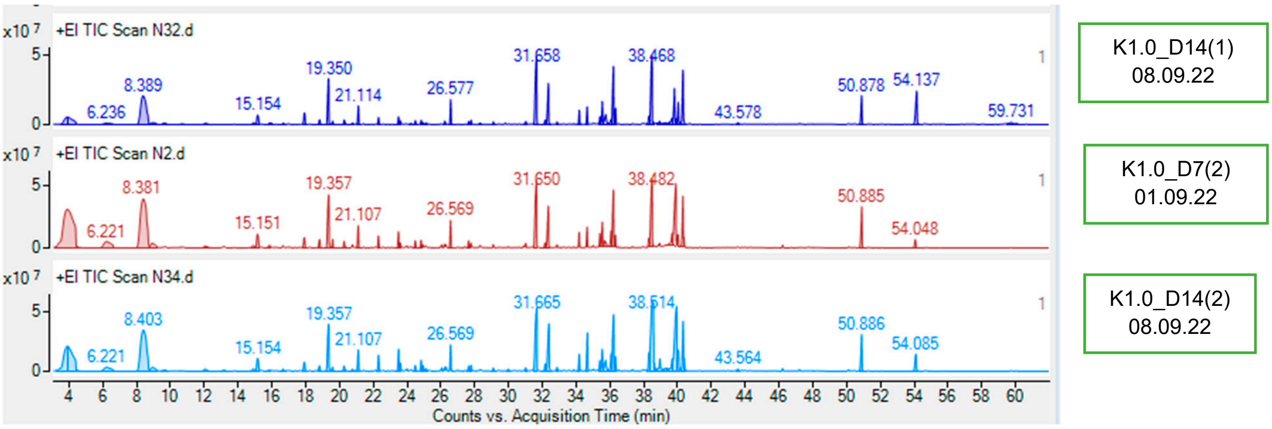

Figure S5. K1.0_D7 and K1.0_D14 chromatograms.

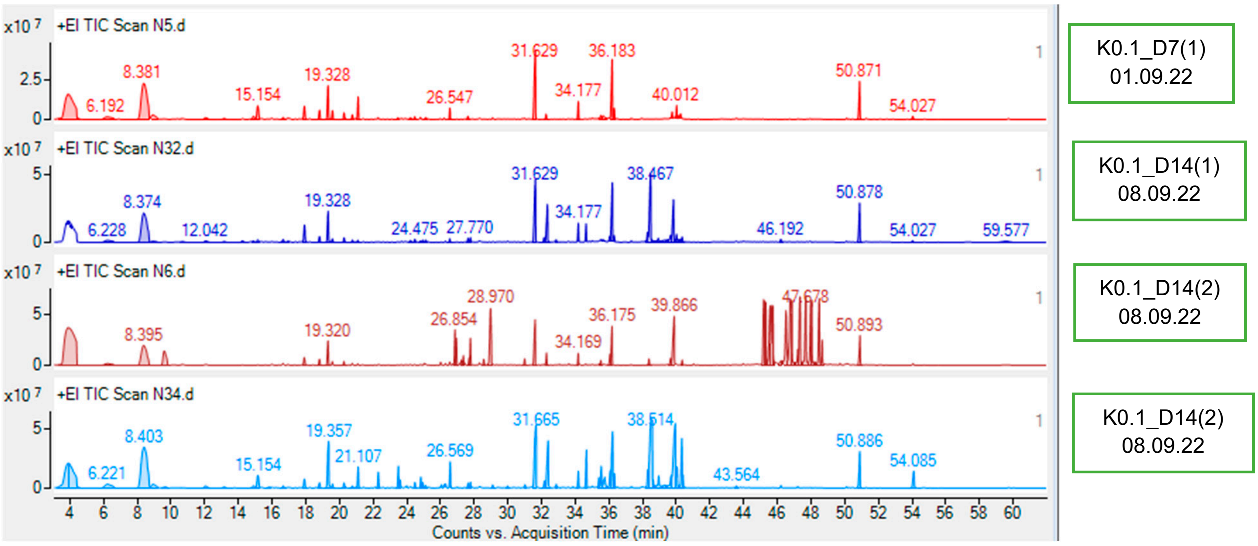

Figure S6. K0.1_D7 and K0.1_D14 chromatograms.

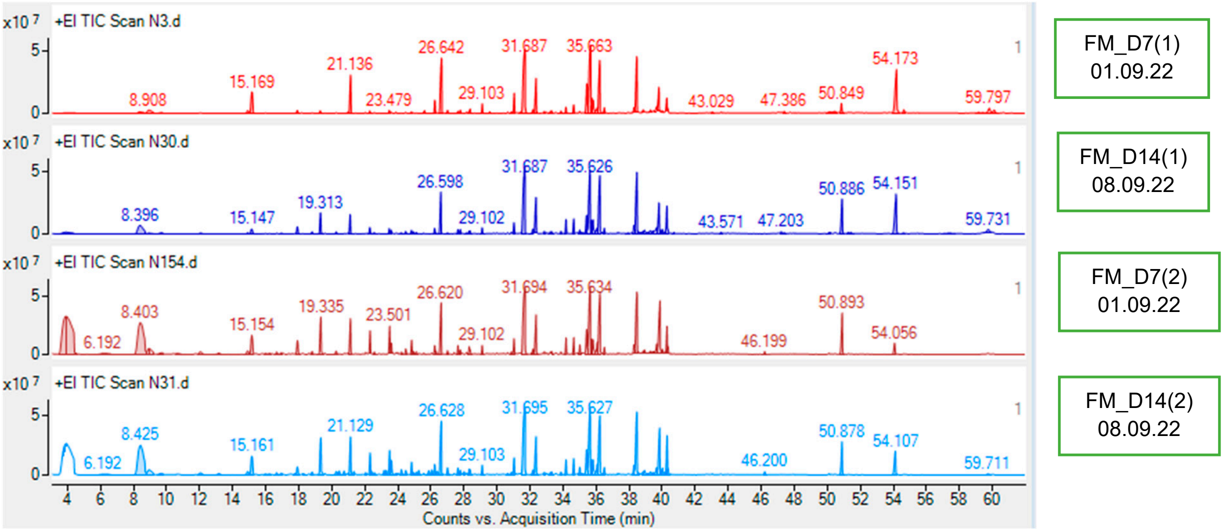

Figure S7. FM_D7 and FM_D14 chromatograms

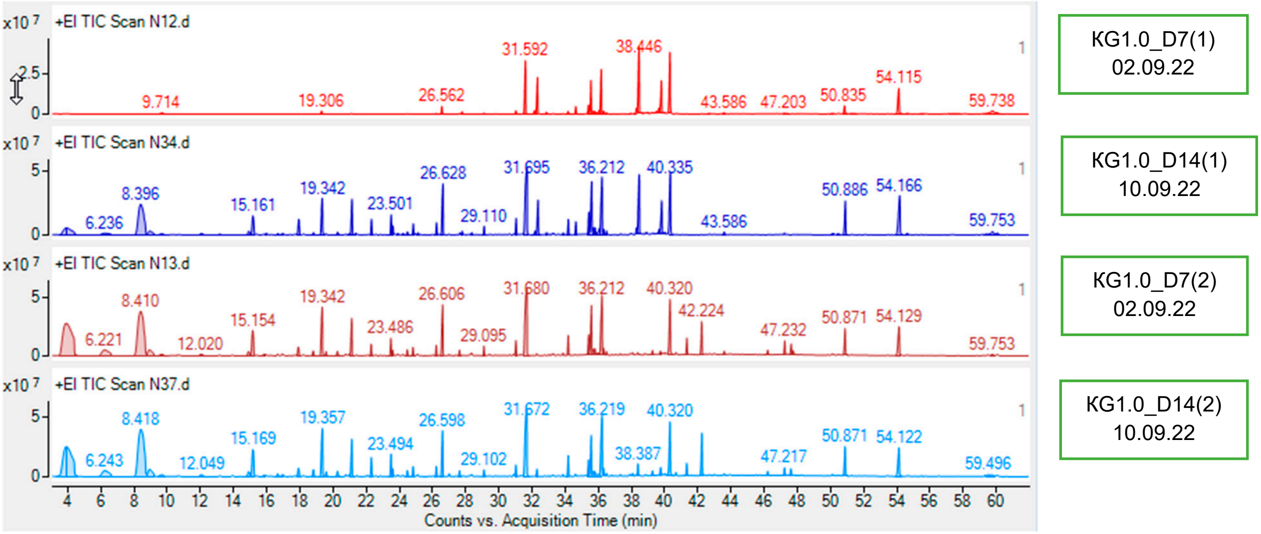

Figure S8. KG1.0_D7 and KG1.0_D14 chromatograms

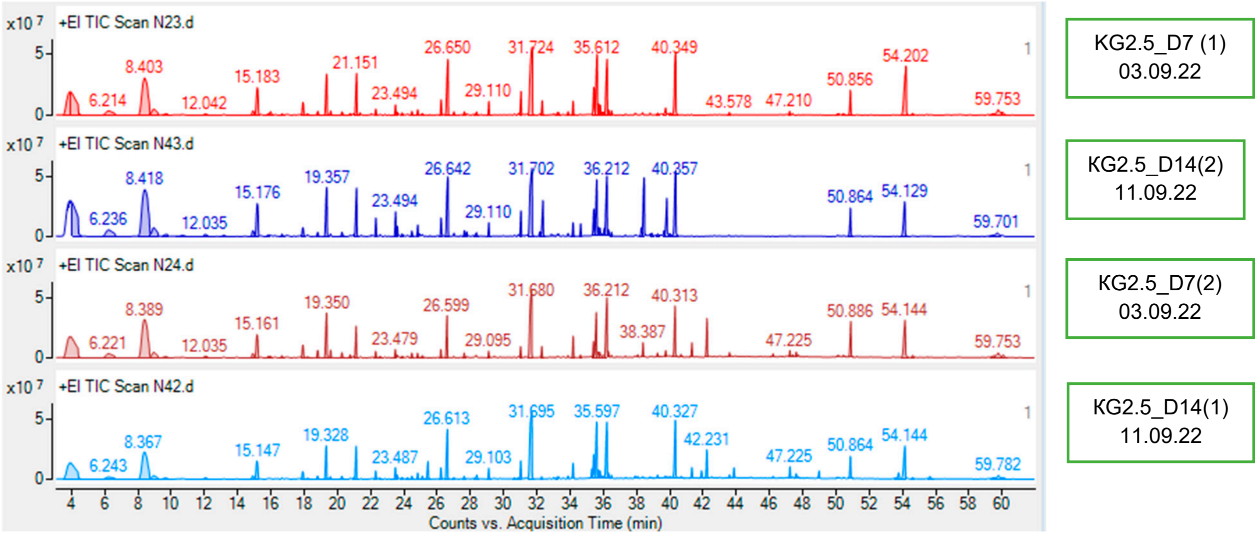

Figure S9. KG2.5_D7 and KG2.5_D14 chromatograms.

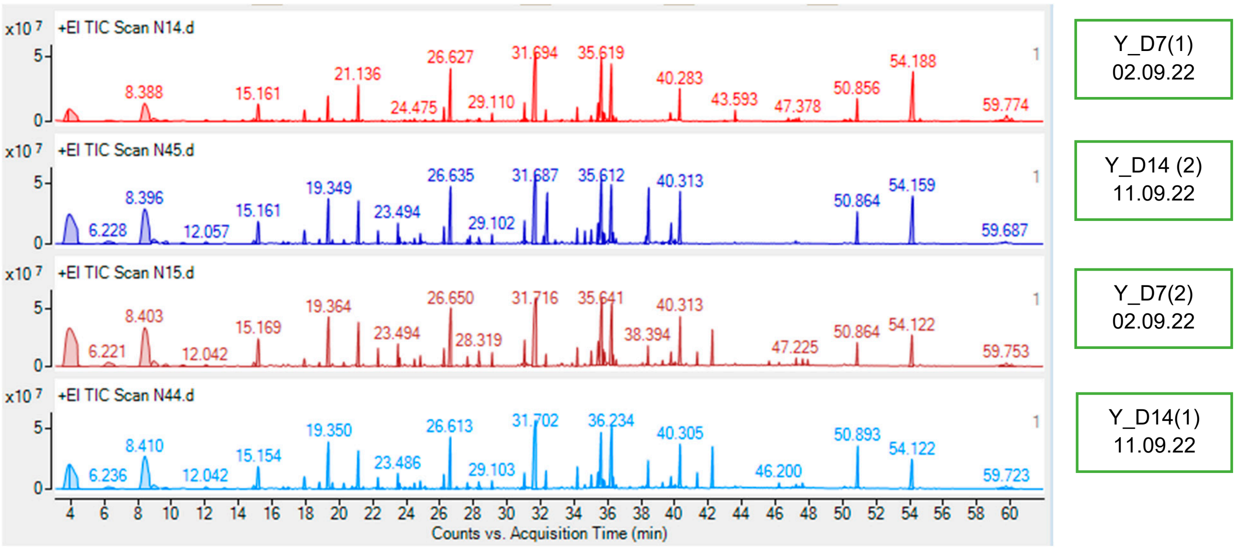

Figure S10. Y_D7 and Y_D14 chromatograms.

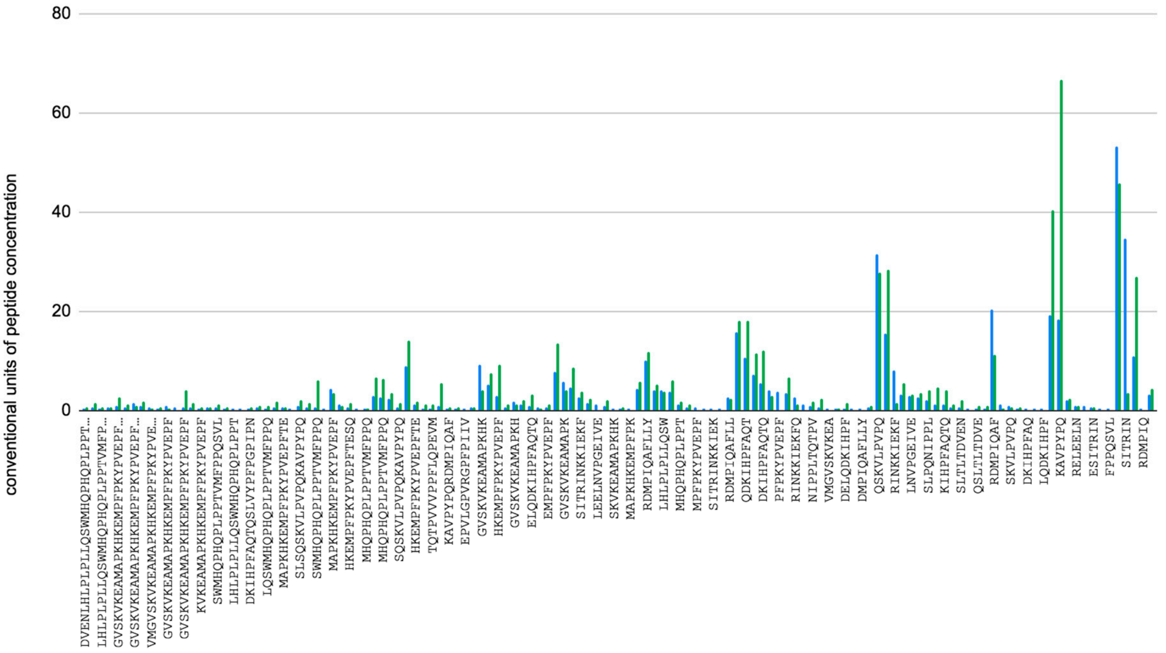

Figure S11. Concentration of peptides of beta-casein origin. KG2.5_D7 – green colour bars; KG2.5_D14 - blue colour bars.

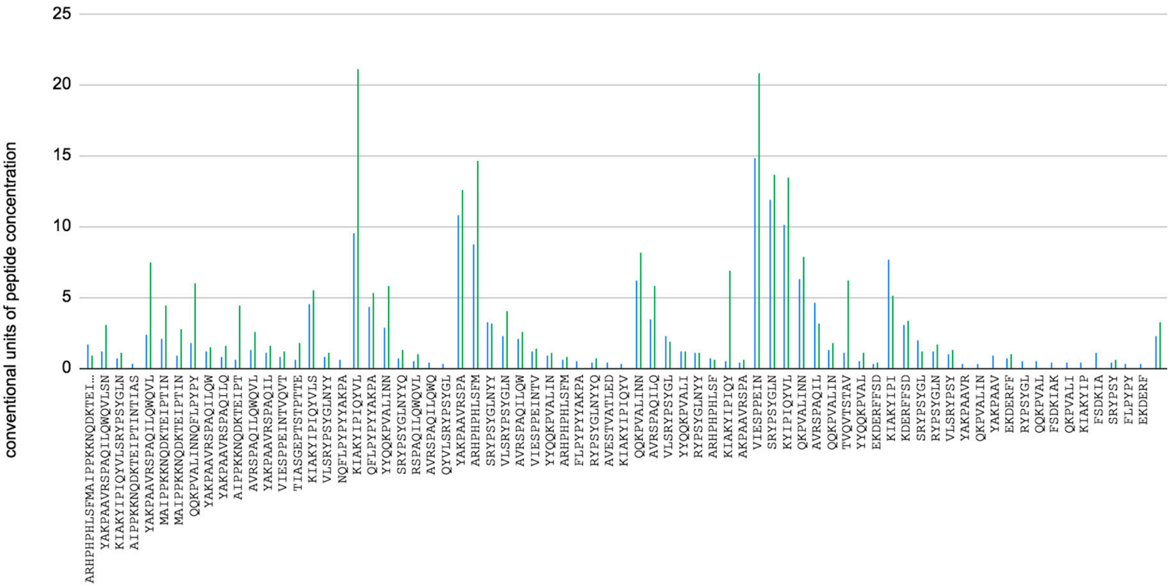

Figure S12. Concentration of peptides of kappa-casein origin. KG2.5_D7 – green colour bars; KG2.5_D14 - blue colour bars.

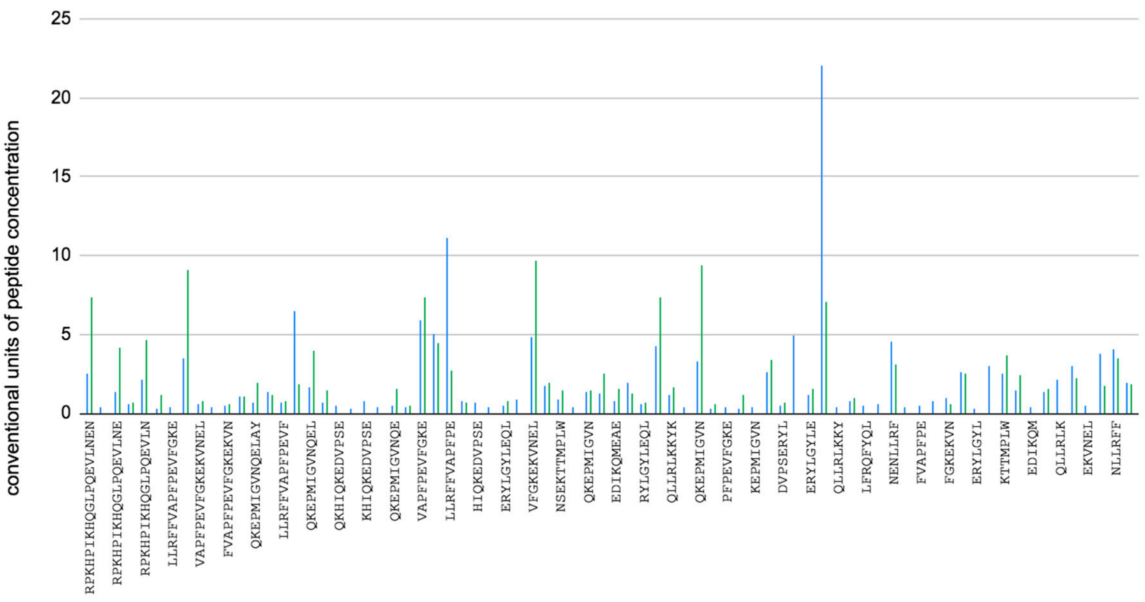

Figure S13. Concentration of peptides of alpha-S1-casein origin. KG2.5_D7 – green colour bars; KG2.5_D14 - blue colour bars.

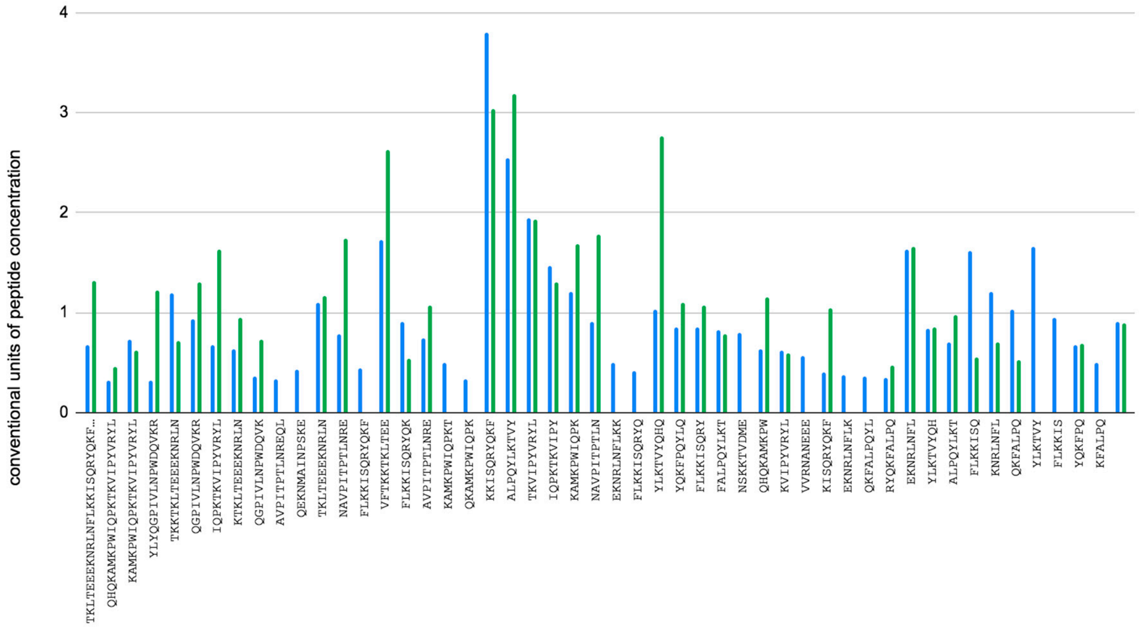

Figure S14. Concentration of peptides of alpha-S2-casein origin, KG2.5_D7 – green colour bars; KG2.5_D14 - blue colour bars.

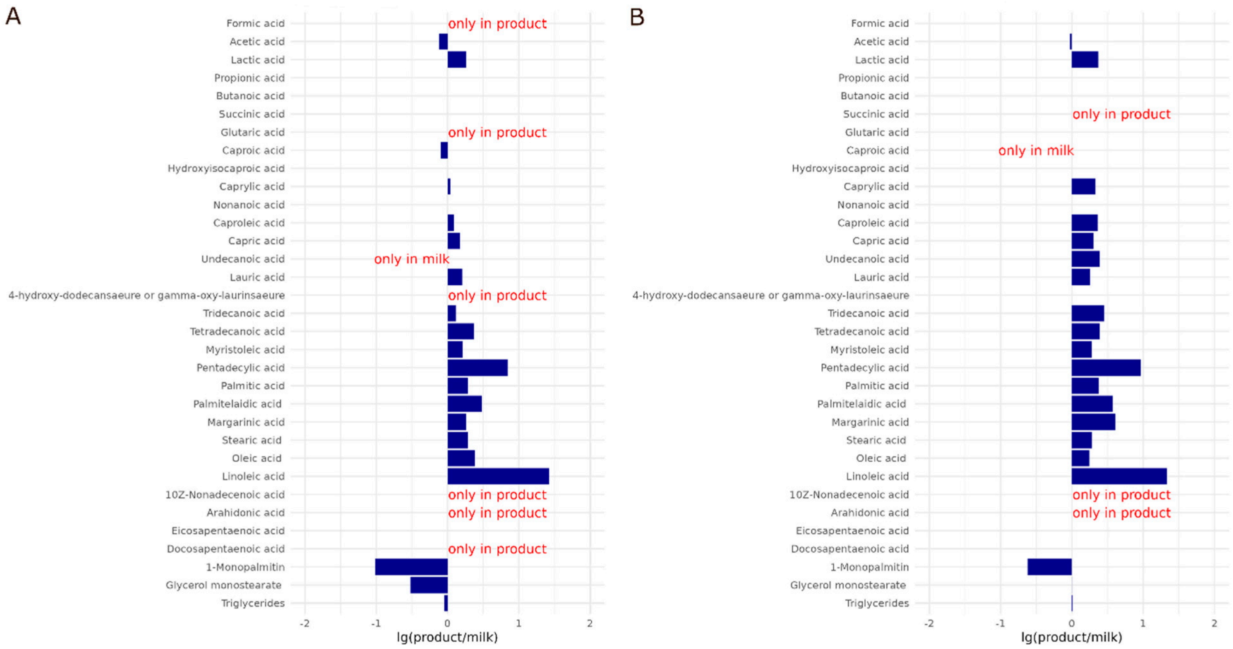

Figure S15. Comparison of medium and long chain fatty acids content in 2.5% fat products with 2.5% milk (M2.5): (A) FM_D7; (B) KG2.5_D7

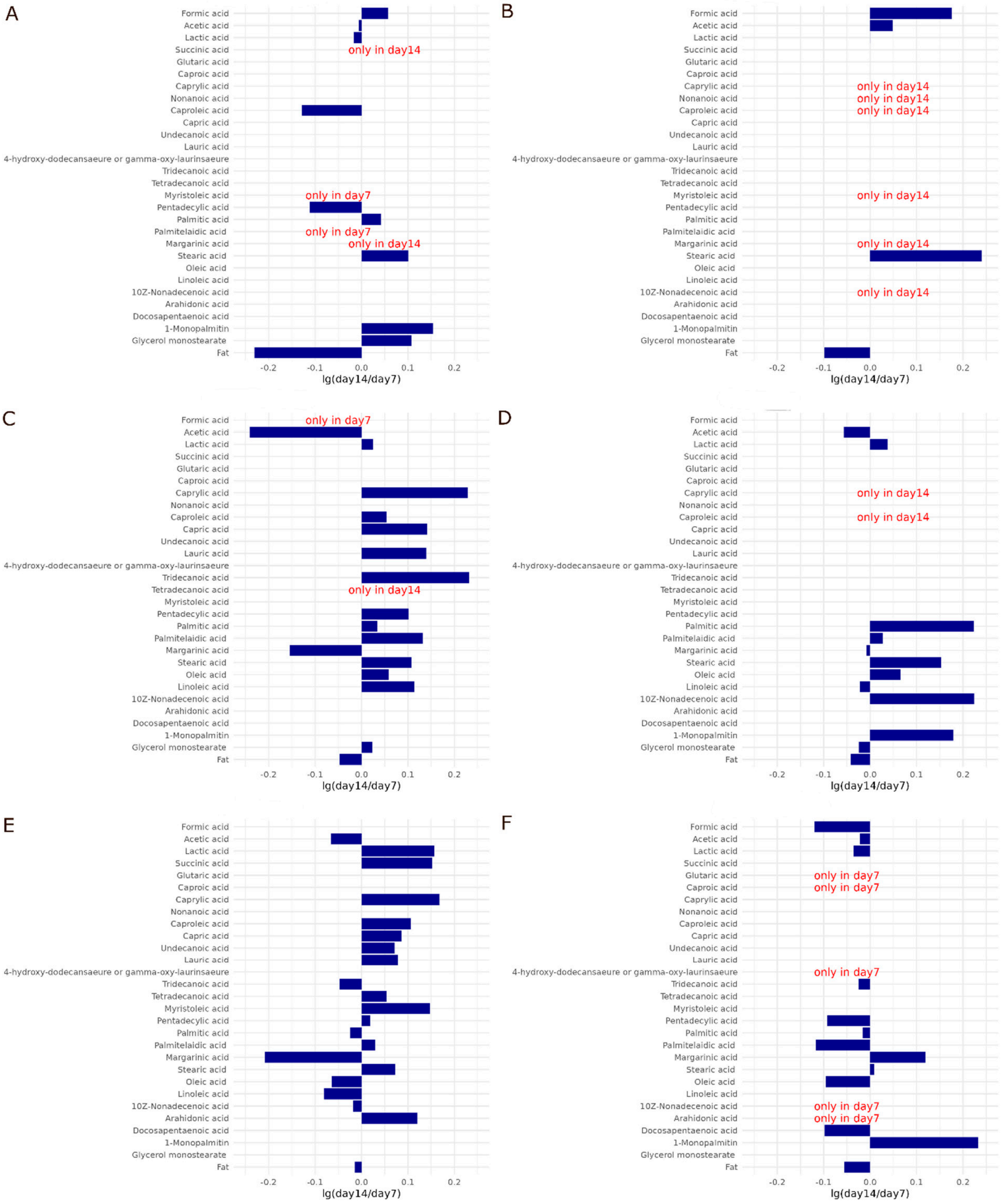

Figure S16. Comparison of medium and long chain fatty acids content in studied products in 7 and 14 days of storage for (A) K0.1; (B) K1.0; (C) Y; (D) KG1.0; (E) KG2.5; (F) FM
